# Computational Study of C-X-C Chemokine Receptor (CXCR)3 Binding with Its Natural Agonists Chemokine (C-X-C Motif) Ligand (CXCL)9, 10 and 11 and with Synthetic Antagonists: Insights of Receptor Activation towards Drug Design for Vitiligo

**DOI:** 10.3390/molecules25194413

**Published:** 2020-09-25

**Authors:** Giovanny Aguilera-Durán, Antonio Romo-Mancillas

**Affiliations:** 1 Posgrado en Ciencias Químico Biológicas, Facultad de Química, Universidad Autónoma de Querétaro, Cerro de las Campanas S/N, Querétaro 76010, Mexico; giovanny.aguilera@uaq.mx; 2 Laboratorio de Diseño Asistido por Computadora y Síntesis de Fármacos, Facultad de Química, Universidad Autónoma de Querétaro, Centro Universitario, Querétaro 76010, Mexico

**Keywords:** CXCR3, CXCL9, CXCL10, CXCL11, protein homology modeling, molecular dynamics

## Abstract

Vitiligo is a hypopigmentary skin pathology resulting from the death of melanocytes due to the activity of CD8^+^ cytotoxic lymphocytes and overexpression of chemokines. These include CXCL9, CXCL10, and CXCL11 and its receptor CXCR3, both in peripheral cells of the immune system and in the skin of patients diagnosed with vitiligo. The three-dimensional structure of CXCR3 and CXCL9 has not been reported experimentally; thus, homology modeling and molecular dynamics could be useful for the study of this chemotaxis-promoter axis. In this work, a homology model of CXCR3 and CXCL9 and the structure of the CXCR3/Gα_i/0_βγ complex with post-translational modifications of CXCR3 are reported for the study of the interaction of chemokines with CXCR3 through all-atom (AA-MD) and coarse-grained molecular dynamics (CG-MD) simulations. AA-MD and CG-MD simulations showed the first activation step of the CXCR3 receptor with all chemokines and the second activation step in the CXCR3-CXCL10 complex through a decrease in the distance between the chemokine and the transmembrane region of CXCR3 and the separation of the βγ complex from the α subunit in the G-protein. Additionally, a general protein–ligand interaction model was calculated, based on known antagonists binding to CXCR3. These results contribute to understanding the activation mechanism of CXCR3 and the design of new molecules that inhibit chemokine binding or antagonize the receptor, provoking a decrease of chemotaxis caused by the CXCR3/chemokines axis.

## 1. Introduction

Vitiligo is a hypopigmentary skin autoimmune pathology characterized by the development of lesions lacking melanin, which is the natural pigment of the skin produced by melanocytes. Vitiligo affects 0.5–2% of the global population, with the highest incidence rates occurring in India, Japan, and Mexico [1,2,3]. This pathology can be classified as either nonsegmental, due to the symmetrical arrangement of the lesions, or segmental, due to the nonsymmetrical disposition of the lesions [4]. Vitiligo is not a fatal pathology; however, the patient’s quality of life is drastically diminished due to social reasons such as discrimination, social isolation, and depression [5,6,7,8,9]. Current treatments for vitiligo are focused on the promotion of repigmentation through the stimulation of melanogenesis and decreased immune system activity and oxidative stress. Unfortunately, these treatments are not totally effective and have limitations, such as the time of treatment (phototherapy), risk of side effects (corticosteroids), and high cost (topical calcineurin inhibitors such as pimecrolimus and tacrolimus) [10,11].

The etiology of vitiligo is not completely understood; nevertheless, different theories could explain the development and progression of this pathology. Amongst these, oxidative stress and the involvement of the immune system are positioned as the most accepted theories, in addition to genetic factors that predispose individuals to vitiligo [3,12,13]. An increase in reactive oxygen species that cause DNA damage, the release of exosomes with intracellular peptides and misfolded proteins, and apoptosis have been reported. Thus, released peptides and misfolded proteins can be recognized by the immune system and stimulate the development of autoreactive lymphocytes responsive to melanocyte epitopes [3,14,15]. Moreover, CD8^+^ T lymphocytes were identified as being responsible for the progress and intensity of vitiligo; once melanocyte antigens are recognized, these lymphocytes release granzyme and perforin, promoting apoptosis in melanocyte cells. Additionally, the proinflammatory process triggers structural damage in the epidermis. All of these factors contribute to the development of new vitiligo lesions [16]. Furthermore, an increase in the concentration of Interferon-γ-dependent chemokines and their respective receptors, which are related to the progression of the pathology, have been observed in patients with vitiligo [11]. When the CD8^+^ T lymphocytes are activated by antigen recognition, the chemotaxis of new cells of the immune system is promoted to the site of damage [17].

Among the factors involved in the chemotaxis process, the most important are the chemokines, which are low molecular weight proteins (between 8–12 kDa) that promote immune cell migration to the site of injury when interacting with their receptor [18]. In patients with vitiligo, the overexpression of chemokine receptors of the CXC family has been reported, namely CXCR3 and CXCR6, as well as the chemokines CXCL9, 10, and 11, natural ligands of CXCR3, and CXCL16, the only known natural ligand of CXCR6 [11,19]. In the case of the CXCR3 axis, the overexpression of the CXCR3 receptor in CD8^+^ and memory T lymphocytes in the skin and peripheral blood of patients with vitiligo has been reported. Moreover, the reduction in CXCL10 and CXCR3 concentrations decreases the lymphocyte infiltration in areas of injury in animal models. Therefore, CXCL10 is considered an important marker in the progression of vitiligo due to the lower overexpression in patients with stable vitiligo than the overexpression in patients with active vitiligo. Additionally, it is considered that stressed keratinocytes are responsible for the production and release of CXCL10 to recruit lymphocytes into the epidermis and promote the melanocytotoxic activity of CD8^+^; in comparison, the inhibition of CXCR3 by antibodies decreases the presence of CD8^+^ lymphocytes in lesions in animal vitiligo models [20,21,22].

The CXCR3 chemokine receptor is a transmembrane G-protein-coupled receptor (GPCR) with three isoforms, CXCR3A (342 aminoacids, a.a.), CXCR3B (451 a.a.), and CXCR3-alt (267 a.a.), by alternative splicing [23,24,25]. CXCR3A is the most expressed isoform, followed by CXCR3B; it has been reported in the selective expression of these isoforms in different pathologies such as cancer, endometriosis, and Crohn’s disease [24,25]. The natural ligands for CXCR3 are chemokines CXCL9 (MIG), CXCL10 (IP-10), and CXCL11 (I-TAC) [26]. These chemokines have different affinities with the receptor; CXCL11 has the highest affinity, followed by CXCL10 and CXCL9. CXCR3A is a GPCR coupled with a G-protein (GP) Gα_i/0_ subunit and its natural ligands, and this isoform is overexpressed in different pathologies of an inflammatory nature, in addition to several types of cancer [27,28], and its activation promotes the mobilization of calcium in the cell, resulting in the chemotaxis of immune system cells [28]. In contrast, CXCR3B is coupled to the Gα_s_ subunit and β-arrestin signaling [23,24,25], and the expression of CXCR3B in the melanocytes of patients with vitiligo is related to the apoptosis of melanocytes in early stages of the pathology by the stimulus of CXCL10 excreted by innated lymphoid cells [29].

In the chemokine–receptor interaction, a “two-site” model for complex formation has been proposed: the receptor first recognizes the binding site located in the N-loop region (“docking domain”) after two cysteines in chemokines. This initial contact facilitates the subsequent binding and proper positioning of the flexible N-terminal region (“triggering domain”), which activates the receptor by interacting presumably with multiple receptor helical sites and inducing a change in the receptor conformation [30]. Furthermore, recent reports propose that there is an alternative binding site to the two-site model, reported in an extensive study of the interaction between CXCL12 and CXCR4 that confirms the relevance of sulfation on tyrosines in CXC receptors, thus expanding the knowledge of chemokine–CXCR interactions [31].

**Figure 1:**
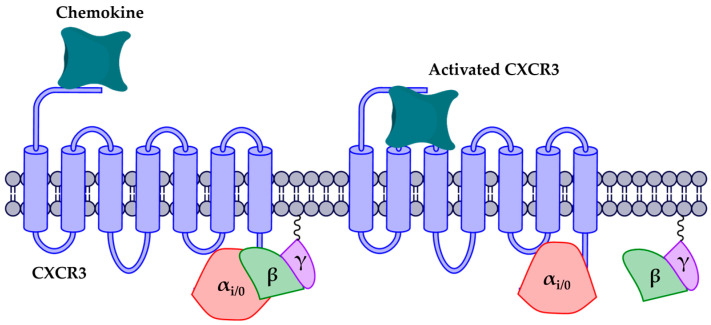
CXCR3 receptor activation. The first activation step is the arrival of the chemokine and the subsequent orientation towards the transmembrane region. The second step is the release of the βγ complex caused by the conformational change of the receptor.

As for activation, it has been proposed that chemokine receptors are activated in two steps: (1) the interaction of chemokine with the extracellular region and the movement into the transmembrane region, promoting a conformational change in the receptor, and (2) separation of the βγ complex from the α_i/0_ subunit (Figure 1) [32,33,34]. Moreover, GPCR activation is related to changes in the rotation angle and polar interactions between the transmembrane segments (TMs). In the rhodopsin receptor (Rho), activation has been reported by the rotation of TMs 1, 3, 5, 6, and 7, where relevant residues such as W265 allow the rotation of TM6 for Rho activation. Similarly, the highly conserved DRY motif is relevant to the stabilization of active and inactive states. This motif contains an ionic interaction between D and R, known as the “arginine cage” [35,36], which is related to an inactive state of the receptors; when the receptor is activated, this interaction breaks down and promotes interactions with other TMs and with the alpha subunit of the G-protein. The DRY motif is present in most chemokine receptors, with the exception of CXCR6, which has an analogous DRF motif [36,37,38,39,40,41].

Synthetic antagonists for CXCR3 have been reported in recent years, mostly found by high-throughput screening (HTS) efforts and subsequent optimization methods [42,43,44,45,46,47,48,49,50,51]; however, studies to understand the mechanism of antagonist and natural ligand binding are limited, because the crystallographic structure of this receptor has not been resolved experimentally [26]. Protein homology modeling is a computational technique that allows an approximation to the three-dimensional structure of a protein that does not have an experimentally resolved tridimensional structure, based on the principle of “similar primary sequences will have a similar three-dimensional conformation”. Thus, the accuracy of the models depends on the similarity of the amino acid sequences to proteins whose crystallographic structures are reported in databases such as the Protein Data Bank [52]. CXCR3 homology models have been reported to elucidate the mechanism of receptor activation and interactions with their chemokines and antagonists; however, these models did not consider post-translational modifications contained in the receptor or were modeled using a template with a low percentage of identity [26,53].

Due to the lack of efficient therapeutic options, the search for new active compounds is necessary to improve the quality of life of patients with vitiligo. The antagonism of CXCR3 to inhibit chemotaxis is a target of interest in the search for new bioactive molecules due to a critical role in the recruitment of CD8^+^ melanocytotoxic lymphocytes in the skin; however, the lack of a three-dimensional structure limits the development of new specific drugs targeted to CXCR3. In this work, the homology modeling of the chemokine receptor CXCR3 and the chemokine CXCL9 is reported; additionally, the molecular dynamics study of interactions between CXCR3 and their chemokines CXCL9, CXCL10, and CXCL11 are presented. The aim of the study was to observe the conformational changes of CXCR3 in the presence of its natural agonists, in addition to the protein–protein interactions present in these complexes, to better understand the two-step activation of this receptor and the interactions of the known antagonists with those conformations. The results could contribute to the future design of new specific antagonists in the therapy of vitiligo and other pathologies in which the CXCR3 axis is involved.

## 2. Results and Discussion

### 2.1. Protein Structures, Homology Modeling, and Relaxation by Molecular Dynamics (MD) Simulations

#### 2.1.1. Protein Structures and Homology Modeling

All Ramachandran plots are shown in the Supplementary Information (Figures S1–S3). The I-TASSER server built five models of CXCR3 using CCR5 as a template (PDB code: 4MBS, chain A), with a sequence identity of 36.49%, considering that a percentage greater than 30% facilitates the prediction of structures at the time of modeling [54]. The best model by I-TASSER was the model1, which was selected by I-TASSER through the evaluation of the C-score and TM-score: model1 had a C-score of -0.65 (with a range of −5 to 2, higher values represent greater confidence in the model) and a TM-score of 0.63 ± 0.14 (greater than 0.5 is considered a model with correct topology). Additionally, model1 was evaluated using the MolProbity public server [55] to obtain a Ramachandran plot to validate this model. The percentage of residues in favorable areas was 85.52% (Figure S1A); the higher the percentage of residues in the favored regions in the Ramachandran plot, the better the stereochemical quality of the homology models [56]. According to this metric, the CXCR3 model by I-TASSER was a suitable model, even without a relaxation process by MD.

In the case of CXCL9, the I-TASSER server built five models of CXCL9 using CXCL2 as a template (PDB code: 5OB5, chain A), with a sequence identity of 47.14%. The best model was the model1 and had a C-score of -1.11 and a TM-score of 0.58 ± 0.14. The percentage of residues in favorable areas in the Ramachandran plot was 75.25% (Figure S1C), indicating a model that needed relaxation by MD to improve its quality.

Similarly, the α_i/0_ subunit was modeled using the structure of chimeric G-alpha proteins (*Rattus norvegicus* and *Bos taurus*) as a template (PDB code: 3V00), with the sequence identities of 87.89% and 65.92%, respectively. The best model had a C-score of 1.00 and TM-score of 0.85 ± 0.08. The percentage of residues in favorable areas in the Ramachandran plot was 91.19% (Figure S1E) [57].

#### 2.1.2. Relaxation of Homology Models

In the short production MD simulation for equilibration of the homology models, the most representative conformation (cluster1) of the CXCL9 five-ns all-atom molecular dynamics (AA-MD) simulation was aligned with the homology model, with a root mean standard deviation (RMSD) of the α-carbon of 2.675 Å (Figure S1), and the percentage of favored residues of the Ramachandran plot was 89.11%, showing that cluster1 conformation was an appropriate conformation for subsequent studies (Figure S1D). RSMD was used as a measure of change in the system with respect to a starting structure.

**Figure 2:**
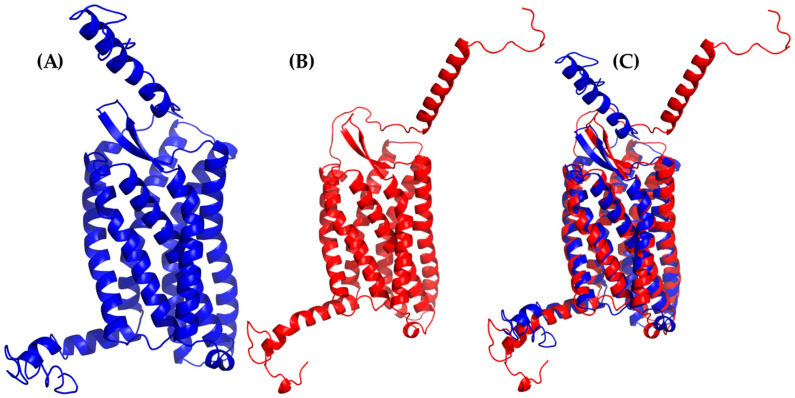
All-atom molecular dynamics (AA-MD) simulation of CXCR3 50 ns. (**A**) CXCR3 model obtained from I-TASSER (initial confirmations or T_0_), (**B**) cluster1 of the simulation, and (**C**) the alignment of T_0_ and cluster1.

Likewise, the alignment of cluster1 from the CXCR3 50-ns AA-MD simulation presented an RMSD of 3.234 Å compared to the homology model obtained by I-TASSER (Figure 2), corresponding to the adjustment of residues that were in unfavorable conformations to improve the protein stability. The percentage of favored residues in the Ramachandran plot increased to 90.44% (Figure S1B), relaxing the conformation to a state of lower energy, thus obtaining a viable model for subsequent studies.

### 2.2. CXCR3-Gα_i/0_βγ Complex Building and Relaxation

Alignment between the 5HT1B (PDB Code: 6G79) and cluster1 from the CXCR3 50-ns AA-MD simulation was made for the addition of Gα_i/0_βγ to the latter. Once the subunits were combined, the resulting complex was evaluated in MolProbity to observe the residues in the favored areas, obtaining 91.91% (Figure S2A). Then, the 100-ns MD simulation was performed to relax the system.

**Figure 3:**
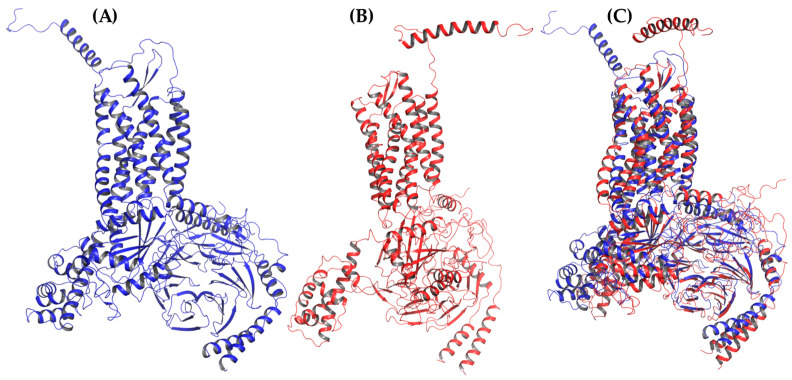
AA-MD simulation of CXCR3-Gα_i/0_βγ 100 ns. (**A**) Structure of the complex at T_0_, (**B**) cluster1 of the simulation, and (**C**) the alignment of T_0_ and cluster1.

Cluster1 of the MD simulation of the CXCR3-Gα_i/0_βγ complex had an RMSD of 5.834 Å compared to the initial confirmations (T_0_) (Figure 3), possibly because the conformation presented by the GP_i/0_ belongs to a stable interaction with the serotonin 5HT1B receptor. The simulation allowed the relaxation of residues to a favorable interaction between the GP_i/0_ and the CXCR3 receptor. After alignment between the GP_i/0_ T_0_ and GP_i/0_ cluster1 of the 100-ns dynamics, a RMSD of 5.022 Å was observed, suggesting a conformational change that promoted the stabilization of the complex.

The alignment between only the CXCR3 receptor was 2.553 Å, showing significant changes in the regions corresponding to the extracellular region, loops, and the intracellular region. Additionally, the Ramachandran plot indicated 92.72% of favored residues (Figure S2B) after the MD simulation of this system. Thus, this structure of CXCR3 was used for building the complexes for subsequent studies of the interaction with CXCL9, 10, and 11.

### 2.3. CXCR3/Chemokines Complex Building and 50-ns AA-MD Simulation

A summary of the clustering of the AA-MD simulations is shown in Table 1.

**Figure 4:**
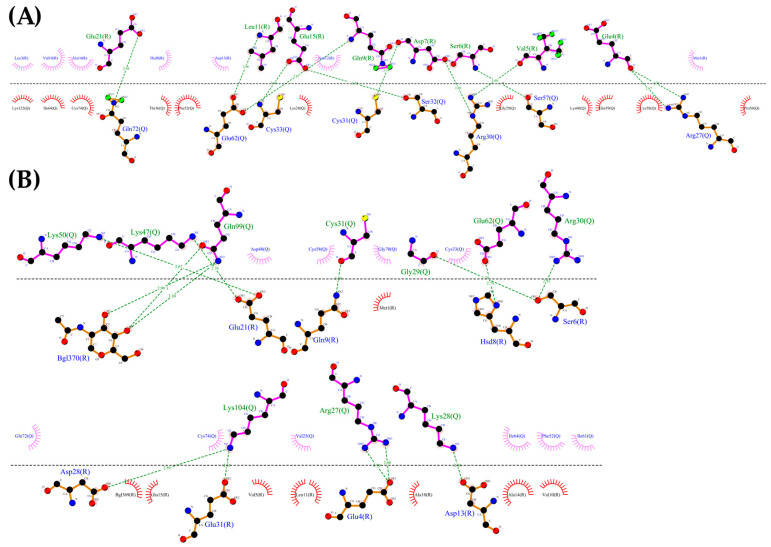
Interactions between CXCL9 and CXCR3. (**A**) CXCR3-CXCL9_T_0_. (**B**) Cluster1_50-ns AA-MD. The R chain corresponds to CXCR3 and the Q chain to CXCL9. An increase of hydrogen bonds in cluster1 of the 50-ns simulation can be observed, as well as an interaction between the Q99 residue of CXCL9 and the glycosylation present in the N32 residue of CXCR3. The residues in red and purple represent hydrophobic interactions between proteins.

**Figure 5:**
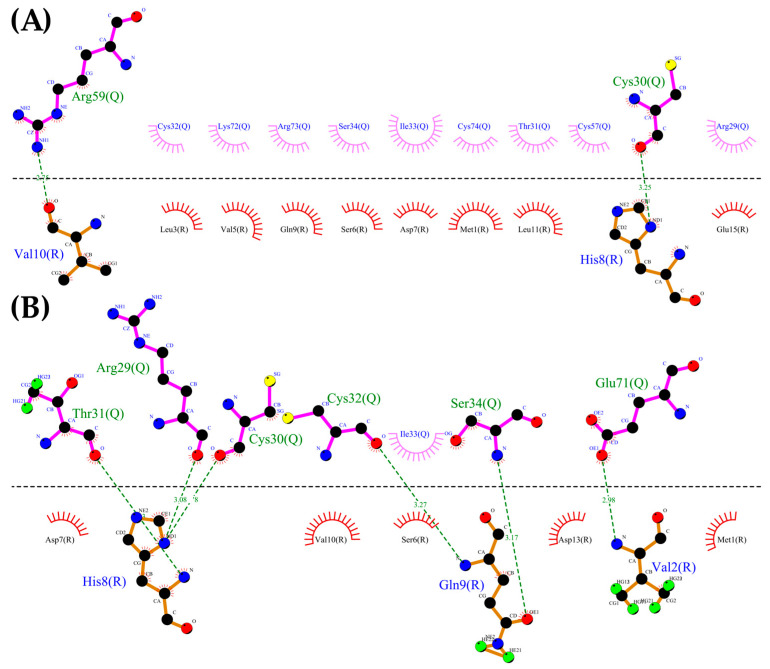
Interactions between CXCR3 and CXCL10. (**A**) CXCR3-CXCL10_T_0_. (**B**) Cluster1_50 ns. The R chain corresponds to CXCR3 and the Q chain to CXCL10. An increase of hydrogen bonds can be observed, along with a decrease of the hydrophobic environment involved and an increase in CXCL10 residues that interact with CXCR3 in cluster1 of the 50-ns simulation.

**Figure 6:**
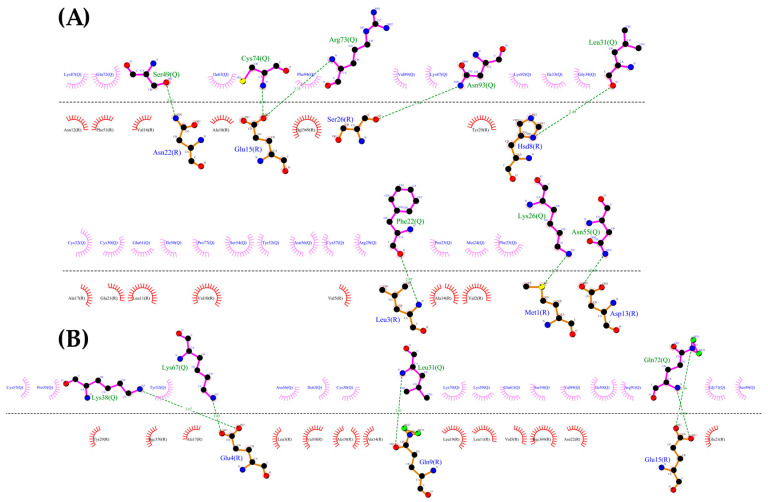
Interactions of CXCR3 and CXCL11. (**A**) CXCR3-CXCL11_T_0_. (**B**) Cluster1_50 ns. The R chain corresponds to CXCR3 and the Q chain to CXCL11. A decrease of interactions between CXCR3 and CXCL11 is observed. Glycosylation in the N22 participates in the hydrophobic environment for the interaction of CXCL11 T_0_ and cluster1.

Using the CXCR3 conformation in cluster1 of the CXCR3-Gα_i/0_βγ complex, the protein–protein dockings were made in the ClusPro server to observe the protein–protein interactions preliminarily between CXCR3 and the different chemokines and positioning the CXCR3/chemokines complex for the MD simulations. The docking was guided by the selected constrains in ClusPro (first 16 residues, Y27, and Y29 in CXCR3) derived by experimental mutagenesis data (see Materials and Methods). The conformation representative of the highest number of conformations in the cluster based on the combination of hydrophobic and electrostatic interactions given by ClusPro was chosen for the AA-MD and coarse-grained molecular dynamics (CG-MD) simulations. These complexes presented a good percentage of residues in favored areas of the Ramachandran plot: 91.60%, 90.87%, and 90.46%, respectively (Figure S3A–C). Once the complexes were built, a 50-ns AA-MD simulation was performed; the interactions between CXCR3 and the chemokines in T_0_ and cluster1 are presented in Figure 4, Figure 5 and Figure 6. All observed salt bridges of the CXCR3–chemokine interactions are included in the Supplementary Information as tables (Tables S1–S5). The CXCR3-CXCL9 complex has two salt bridges and the CXCR3-CXCL10 complex has one salt bridge; in contrast, the CXCR3-CXCL11 complex does not have salt bridges (Table S1). No hydrogen bonds were made with the post-translational modifications included in CXCR3 in any complex. Additionally, for CXCR3-CXCL9, cluster1 had a hydrogen bond between Q99 and glycosylation in the N32 residue of CXCR3 (Figure 4B). By comparison, CXCL10 and CXCL11 did not show interactions with glycosylations, showing the importance of post-translational modifications for the interaction between CXCL9 and CXCR3 and disregarding their relevance in the initial interaction of CXCL10 and CXCL11; however, in cluster1 of CXCR3-CXCL11, the residue N22 participates in the hydrophobic environment, promoting the interactions between CXCR3 and CXCL11 (Figure 6B). The salt bridges between CXCR3 and the chemokines had an interesting change: in the CXCR3-CXCL9 complex, they increased to a total of nine, while there were none in CXCR3-CXCL10 and two in CXCR3-CXCL11 (Table S2). Residues with post-translational modifications were not involved in any of the cases.

**Figure 7:**
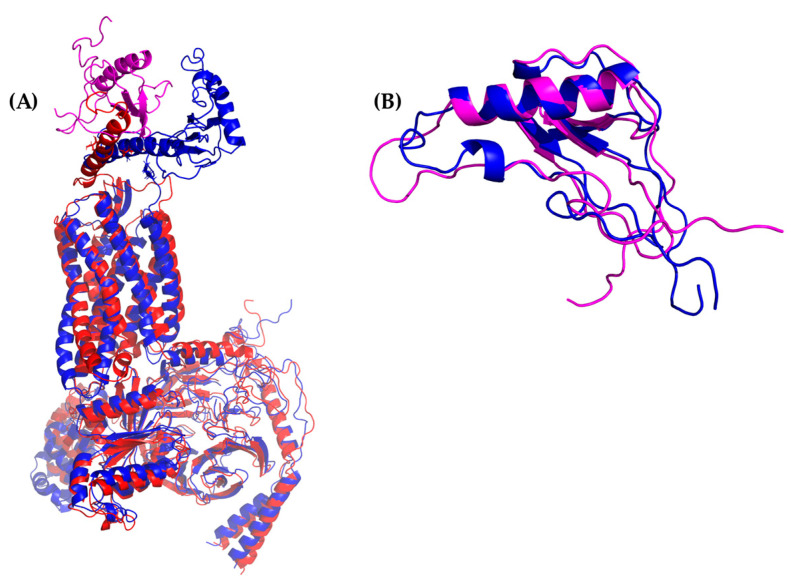
AA-MD simulation of CXCR3-CXCL9. (**A**) Alignment of CXCR3-CXCL9_T_0_ (blue) and CXCR3-CXCL9_50 ns (red). (**B**) Alignment of CXCL9_T_0_ (blue) and CXCL9_50 ns (magenta). There is a root mean standard deviation (RMSD) of 2.841 Å between T_0_ and cluster1 of the 50-ns simulation, and changes in the Gα_i/0_βγ are observed; in addition, there is a conformational change of CXCL9 with an RMSD of 2.972 Å, which indicates that the system evolves into a more favorable state for interactions between CXCL9 and CXCR3.

**Figure 8:**
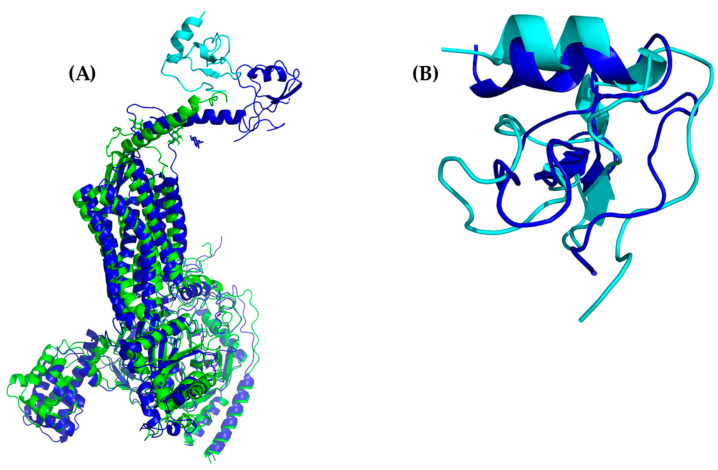
AA-MD simulation of CXCR3_CXCL10. (**A**) Alignment of CXCR3-CXCL10_T_0_ (blue) and CXCR3-CXCL10_50 ns (green). (**B**) Alignment of CXCL10_T_0_ (blue) and CXCL10_50 ns (cyan). There is an RMSD of 2.646 Å between T_0_ and cluster1 of the 50-ns simulation; changes in the Gα_i/0_βγ are observed; additionally, a conformational change of CXCL10 exists with an RMSD of 4.745 Å, which shows that the system evolves into a more favorable state for interactions between CXCL10 and CXCR3.

**Figure 9:**
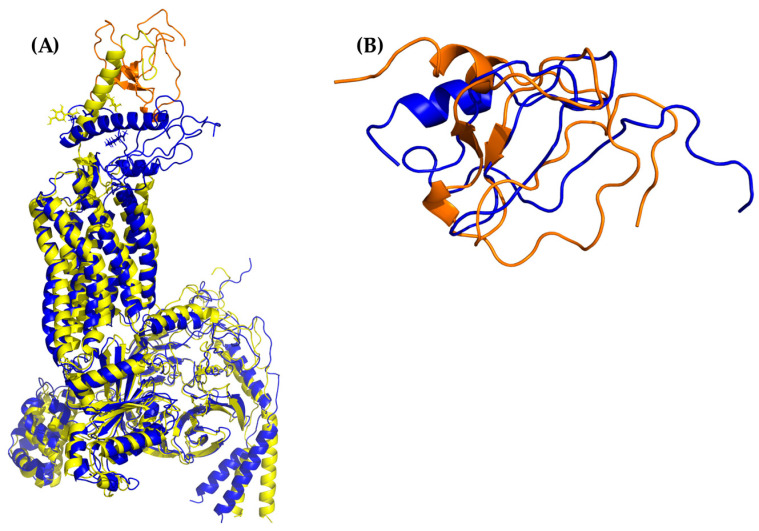
AA-MD simulation of CXCR3_CXCL11. (**A**) Alignment of CXCR3-CXCL11_T_0_ (blue) and CXCR3-CXCL11_50 ns (yellow). (**B**) Alignment of CXCL11_T_0_ (blue) and CXCL11_50 ns (orange). There is an RMSD of 2.538 Å between T_0_ and cluster1 of the 50-ns simulation; changes in the Gα_i/0_βγ are observed in addition to a conformational change of CXCL10 with an RMSD of 6.339 Å; however, unlike CXCL9 and 10, the hydrogen bonds and hydrophobic-type interactions decreased.

Additionally, the binding of chemokines with CXCR3 caused changes in the receptor, with an RMSD of 2.841 Å for CXCL9, 2.646 Å for CXCL10, and 2.538 Å for CXCL11, mainly in the transmembrane region but, also, in Gα_i/0_βγ (Figure 7A, Figure 8A and Figure 9A). The comparison of the initial conformations (T_0_) and the representative clusters shows a considerable change, which indicates that the system conformationally evolves to find a more stable interaction between the different chemokines and CXCR3. CXCL11 presents a considerable change, with an RMSD of 6.339 Å; however, interactions with CXCR3 are decreased compared to CXCL9 and CXCL10 (Figure 4B, Figure 5B and Figure 6B).

Once the interactions and evolution of the CXCR3-chemokine systems were observed, it was expected that the two-sites model interactions were present in the complexes. In the case of the CXCL9-CXCR3 complex, the chemokine had interactions at T_0_ in the N-loop region; after 50 ns of simulation, the interactions increased to the other residues of CXCL9, similarly to the cases of CXCL10 and CXCL11. Nevertheless, this simulation time was insufficient, since the first activation step could not be observed, which consists of the approach of chemokines to the transmembrane region of the receptor and the subsequent conformational change of the receptor. Within this time frame, only the first interactions between the chemokines and CXCR3 could be observed.

### 2.4. Complex Stabilization in CG-MD Simulations

To verify the two-step activation of the CXCR3 receptor, CG-MD simulations were performed. Currently, the Martini 2.2p force field implemented on CHARMM-GUI does not have parameters for small molecules and modified residues; thus, the addition of post-translational CXCR3 modifications was not possible. However, CG-MDs allowed larger simulation times with shorter calculation times.

The complexes of CXCR3-Gα_i/0_βγ/chemokines (obtained from docking in ClusPro) were used as a starting structure. According to the results of the CG-MD simulations, the representative clusters were around 500–800 ns, as seen in Table 2.

**Figure 10:**
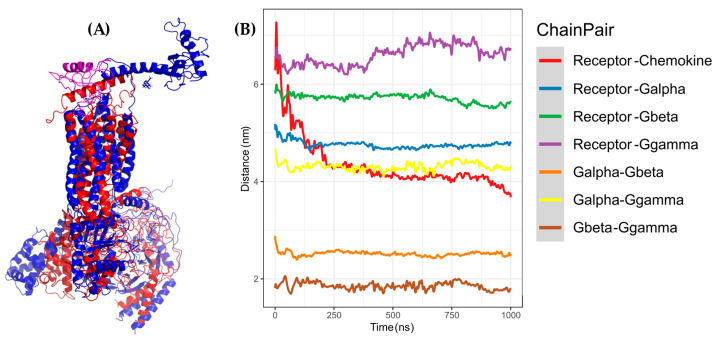
Coarse-grained molecular dynamics (CG-MD) simulation of CXCR3-CXCL9. (**A**) Alignment between CXCR3-CXCL9_T_0_ (blue) and cluster1_CG-MD (red), with an RMSD of 9.409 Å. (**B**) Distances among the CXCR3-CXCL9 complex chains. The distance decreases during the simulation by approximately 2 to 3 Å.

**Figure 11:**
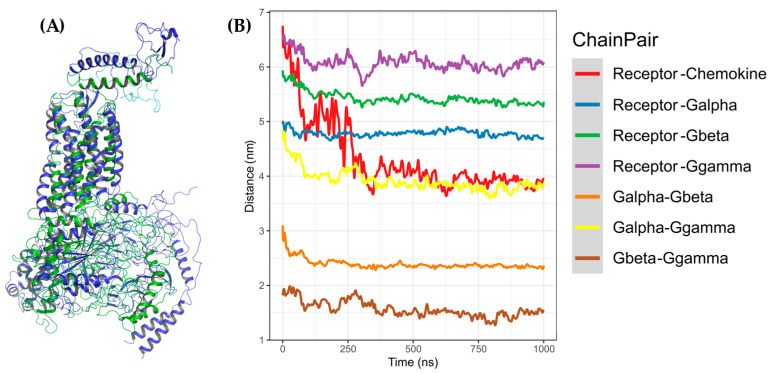
CG-MD simulation of CXCR3-CXCL10. (**A**) Alignment of CXCR3-CXCL10_T_0_ (blue) and cluster1_CG-MD (green), with an RMSD of 9.409 Å. (**B**) Distances among the CXCR3-CXCL10 complex chains. The distance decreases during the simulation by approximately 2 to 3 Å.

**Figure 12:**
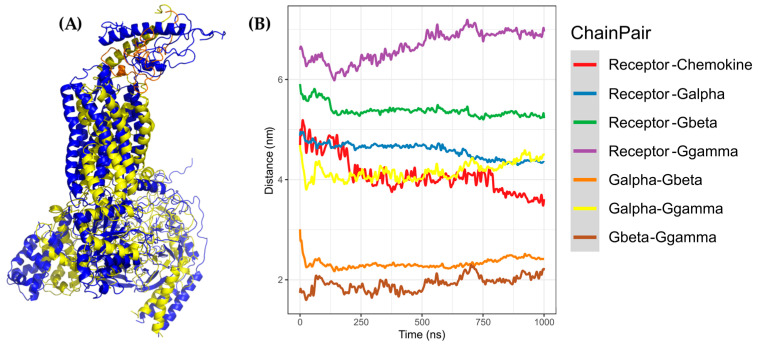
CG-MD simulation of CXCR3-CXCL11. (**A**) Alignment of CXCR3-CXCL11_T_0_ (blue) and cluster1_CG-MD (yellow), with an RMSD of 9.853 Å. (**B**) Distances among the CXCR3-CXCL11 complex chains. The distance decreases during the simulation by approximately 1 Å less than CXCL9 and CXCL10.

In these simulations, the first step of receptor activation could be inferred by the decrease of the distance between the chemokine and the transmembrane region of CXCR3; additionally, a conformational change was observed in the complex, expressed as an RMSD of 9.397 Å for CXCL9, 10.724 Å for CXCL10, and 9.885 Å for CXCL11, with respect to the T_0_ of each simulation. The changes in the extracellular region and Gα_i/0_βγ in the representative clusters of CG-MD could be detected visually. Moreover, there was a decrease in the distance between the chemokines and CXCR3 proportional to the simulation time (Figure 10, Figure 11 and Figure 12).

**Figure 13:**
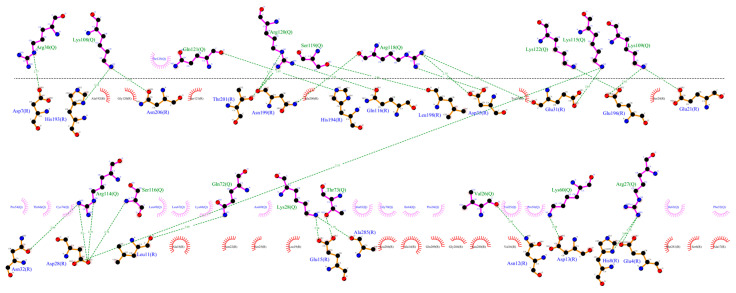
Interactions between CXCR3-CXCL9 from the CG-MD simulation. There is an increase in hydrogen bonds and hydrophobic interactions of CXCR3 with CXCL9 after the CG-MD simulation.

**Figure 14:**
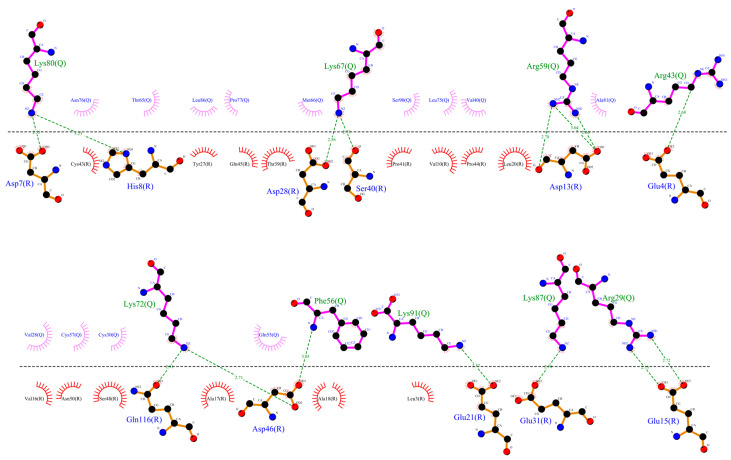
Interactions between CXCR3 and CXCL10 from the CG-MD simulation. An increase in hydrogen bonds and hydrophobic interactions is observed, although, in comparison with CXCL9, the number of polar interactions is lower. The interactions are maintained with the first residues of CXCR3.

**Figure 15:**
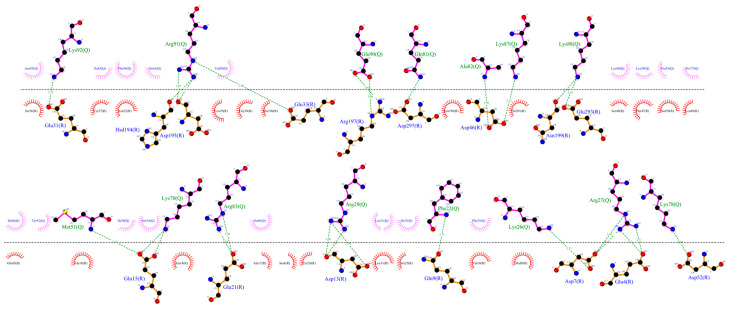
Interactions between CXCR3-CXCL11 from the CG-MD simulation. An increase of hydrogen bonds and hydrophobic interactions is observed. The diagram shows the interaction with the R197 residue of CXCR3, a residue reported to be important for the binding of CXCL11, and interactions with E293, which is reported to be necessary for the stabilization of the CXCL11-CXCR3 complex.

Furthermore, the interactions between CXCR3 and the chemokines in the representative clusters of CG-MD simulations were calculated. For both CXCL9 and CXCL11, the hydrogen bonds and hydrophobic interactions increased significantly, whereas CXCL10 did not show a large increase in the number of polar interactions; however, the number of hydrophobic interactions increased with respect to T_0_ or cluster1 of the 50-ns AA-MD simulation (Figure 13, Figure 14 and Figure 15).

Interestingly, CXCR3 residues involved in the interaction with the chemokines were in the extracellular loops and near the transmembrane region. For CXCL9, there were no coincidences of direct interactions with the relevant residues considered for protein–protein docking (see Materials and Methods). However, they were close to residues that form the CXCR3-CXCL9 interaction observed in the CG-MD simulation; specifically, these were the residues N22 and Y27, which form hydrophobic interactions, and those that should have post-translational modifications (glycosylation and sulfation). Therefore, it cannot be discarded that these modifications could have a significant influence on the interaction with CXCL9. By comparison, salt bridge-type interactions increased to a total of 15 (Table S3).

In the case of CXCL10, there was also no coincidence between the representative conformations of the CG-MD simulation and the previously reported residues. Additionally, the hydrophobic interactions increased; these interactions are relevant in the activation of CXCR3 by CXCL10, and the interaction with residue Y27 of CXCR3 was detected, suggesting that this could be a significant influence of post-translational modifications in the interaction between CXCL10 and CXCR3. In parallel, there was an increase of 12 salt bridges (Table S4).

In CXCL11, hydrogen bonds are observed with R197 and E293 of CXCR3, which is reported as being important for CXCR3-CXCL11 binding to stabilize the complex. By comparison, the salt bridges increased to a total of 15 (Table S5).

Finally, due to the interactions observed between CXCR3 and the different chemokines, it can be suggested that more residues could be responsible for the conformational change towards the first activation step of the CXCR3 receptor. However, the βγ complex did not separate from the α_i/0_ subunit; on the contrary, they approached each other, possibly because this simulation did not contain the nucleotide necessary for the activation of Gα_i/0_βγ. In general, GPs are in the presence of guanosine-diphosphate (GDP), which promotes an inactive state and changes to an activated state when the nucleotide exchanges from GDP to guanosine triphosphate (GTP) with the subsequent separation of the βγ complex with the α subunit, triggering the signaling process [41]. Nevertheless, the conformational changes present in the transmembrane region in CXCR3 indicated an activation process that corresponded to that expected when the different chemokines interact; in the first timesteps, there was an interaction with the N-terminal region of CXCR3; then, the chemokine was orientated to the transmembrane region of CXCR3 as the simulation progressed towards the active conformation of the receptor.

To verify if the conformational changes of CXCR3 induced by the presence of the chemokines contributed a functional state of the receptor, analyses of the rotation and interactions of the TMs, the presence or absence of the arginine cage, and interactions between each chemokine and CXCR3 every 100 ns were performed. The conformation at 0 ns was considered as a reference; this structure was obtained after the minimization and equilibrium process of the system in the CG-MD simulation. The presence or absence of the interaction between D148-R149 forming the arginine cage, which is related to the activation of the GPCRs, was present in different conformations of the CG-MD simulations (Table 3).

#### 2.4.1. Conformational Changes of the Transmembrane Segments (TMs)

The rotation of TM1, TM3, TM5, TM6, and TM7 was important to confirm the activation of the GPCRs; in addition, the hydrogen bonds among them changed in the presence of agonists and antagonists [39,40,41]. The CXCR3 transmembrane segments, identified by UniProt and considered in this work, are presented in Table 4 [58]. All polar and hydrophobic interactions are provided in the Supplementary Information.

**Figure 16:**
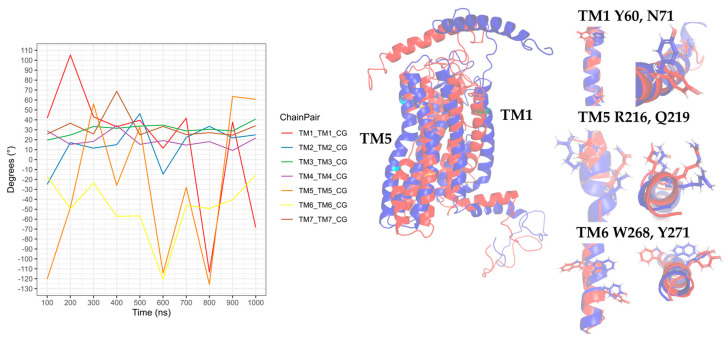
Rotation of the transmembrane segments (TMs) from the CG-MD simulation of CXCR3_CXCL9. The rotations of the TMs throughout the simulation are seen, with intense rotations of TM1, TM5, and TM6. In blue is shown the initial conformation; in red, the conformation at 800 ns.

In the CXCR3-CXCL9 CG-MD simulation, changes in the rotation angles of the TMs were observed. TM2, TM3, TM4, and TM7 were maintained with smaller rotations of TM1, TM5, and TM6. Drastic changes in the TM rotations occurred in the 600–1000-ns range. The rotations of TM5 and TM6 at 600 and 700 ns agreed with the loss and regeneration of the arginine cage, as seen in Figure 16. TM1, TM5, and TM6 presented intense rotations from 600 ns, which corresponded to the loss of the arginine cage in the simulation; however, this interaction was regenerated at 700 and 1000 ns, corresponding to the rotations of TM5 and TM6 at 700 ns and TM1 and TM5 at 1000 ns. The polar interactions between CXCL9 and CXCR3 (Table S6) increased as the simulation progressed, and the most frequent residues were lysine types, agreeing with previous reports on the relevance of positively charged residues in the chemokines for the interaction with CXCR3. The interactions between the TMs increased during the simulation. The residue R216 in TM5 was reported as a relevant residue for CXCR3 activity [59]; in this simulation, R216 was kept in constant interaction between N132 (TM3) and D186 (TM4) (Table S7).

**Figure 17:**
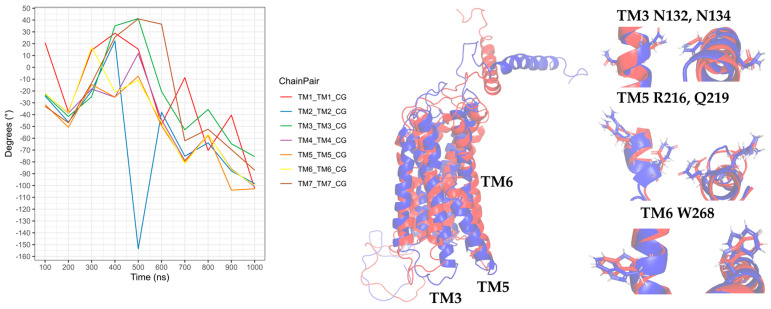
Rotation of the TMs from the CG-MD simulation of CXCR3-CXCL10. The rotations of the TMs throughout the simulation are seen, with intense rotations of TM1, TM2, TM5, and TM6. In blue, the initial conformation is shown; in red is the conformation at 700 ns.

Regarding the CXCR3-CXCL10 complex, the receptor exhibited different behaviors in the presence of CXCL10 compared to the binding of CXCL9, and all the TMs had higher rotations throughout the simulation, as seen in Figure 17. The rotation of TM2 underwent a drastic change at 500 ns and continued with relevant changes during the subsequent nanoseconds; TM1, TM3, and TM7 showed similar behaviors with intense rotations between 300 and 600 ns, and from 500 ns, TM5 and TM6 underwent relevant changes. The greatest changes in the rotation of the TMs occurred after 300 ns, matching with the absence of the arginine cage. Furthermore, after 300 ns, CXCL10 reached an equilibrium in terms of the distance from the center of the mass of CXCR3. The polar and hydrophobic interactions between CXCL10 and CXCR3 (Table S8) increased from 300 ns, and interactions between the R197 residue of CXCR3 and A64 and E71 of CXCL10 were formed at 600 ns. R197 is an important residue for binding CXCL10 and CXCR3 activity.

The interactions between the transmembrane segments were lower compared to the presence of CXCL9; additionally, no interactions of R216 with TM3 and TM4 were present in any of the frames analyzed, but the interactions between N132 and Q219 and D186 and Q219 were maintained (TM3 and TM4 with TM5, respectively). The interaction between D186 and Q219 was maintained practically throughout the simulation; on the contrary, the interaction between N132 and Q219 occurred constantly from 300 ns and was lost at 700 ns, coinciding with the regeneration of the arginine cage. This may suggest that the TM4-TM5 interaction between D186 and Q219 is necessary for CXCR3 to have a stable conformation of the transmembrane segments, and this interaction acts as a stabilization point between the active and inactive states. By comparison, TM2 constantly interacted with TM3, TM4, and TM7 from 300 ns, and the interactions with TM3 and TM4 were lost at 700 ns, coinciding with the regeneration of the arginine cage. Therefore, TM2 is relevant for stabilization of the functional conformation of CXCR3 (Table S9).

**Figure 18:**
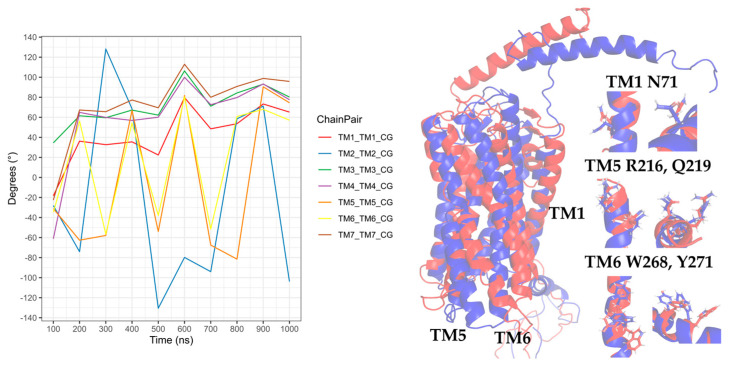
Rotation of the TMs from the CG-MD simulation of CXCR3_CXCL11. Rotations of the TMs throughout the simulation are present, with relevant rotations of TM1, TM2, TM5, and TM6, starting at 200 ns. In blue, the initial conformation is shown; in red is the conformation at 600 ns.

In the simulation of the CXCR3-CXCL11 complex, the CXCR3 transmembrane segments, by the stimulation of CXCL11, rotated with greater intensity compared to the stimulation of CXCL9 and CXCL10 (Figure 18). At 200 ns, the TMs (with the exception of TM3) rotated intensely, concurring with the loss of the arginine cage, and TM3, TM4, and TM7 maintained a similar behavior throughout the simulation without major changes, with a relevant peak at 600 ns. By comparison, TM5 and TM6 presented drastic changes from 200 ns, in agreement with the loss of the arginine cage from 200 ns and regeneration at 400 and 800 ns. TM2 underwent a relevant rotation during the simulation, changing before the regeneration of the arginine cage at 300 and 700 ns. A change in the rotation of the transmembrane segments occurred at 600 ns, where TM1, TM5, and TM6 had greater magnitudes concerning TM2, TM4, TM3, and TM7. Similarly, the polar interactions between CXCL11 and CXCR3 increased from 200 ns (Table S10) but decreased before and during the regeneration of the arginine cage (300, 400, 700, and 800 ns). From 100 ns, interactions existed between R197 and E293 of CXCR3 with residues of CXCL11. These residues are relevant for the binding of CXCL11, and the interaction of R197 was maintained throughout the simulation. In contrast, the interactions with E293 were lost at 500 and 900 ns, matching with the 100-ns conformation after the regeneration of the arginine cage at 400 and 800 ns. This indicates that the behavior of the DRY segment possibly influences the generation of interactions between the chemokine and E293.

Interactions of the transmembrane segments increased from 200 ns. In the case of the stimulation of CXCL11 in CXCR3, the interactions between TM3 and TM5 through N132-Q219 were not present in any of the analyzed time frames. However, interactions between TM3 and TM6 through G138-W268 were observed after 200 ns, and the TM4-TM5 interactions between D186 and Q219 were maintained during the simulation. TM2 showed interactions with TM3, TM4, and TM7 after 200 ns, and there was an interaction between the DRY segment with Y233 of TM5 and interaction between C234 of intracellular loop 3 (Table S11).

Transmembrane segment 2 is relevant for the conformational changes of CXCR3 by the stimulation of CXCL10 and CXCL11 but not of CXCL9. In the presence of CXCL9, R216 in TM5 continued to interact with N132 (TM3) and D186 (TM4); in contrast, with the stimulation of CXCL10 and CXCL11, the interaction between N132 and R216 was not seen during the simulation. The network of interactions between TM3, TM4, and TM5 appears to be necessary for the stabilization of the functional conformation of CXCR3, specifically N132-D186-R216-Q219 for CXCL9 and N132-D186-Q219 for CXCL10 and CXCL11. Interactions between TM3 and TM6 were present due to the stimulation of CXCL11 through G138-W268, concurring with the loss of the arginine cage and the rotation of the transmembrane segments. These results reinforced the fact that the CXCR3 receptor underwent conformational changes that promoted activation in the presence of the chemokines in our simulations, proving the first step of receptor activation of the chemokines.

To support the results of the CXCR3/chemokine CG-MD simulations, a 1-µs CG-MD simulation of the CXCR3/GP complex was performed, and the rotation angles and polar interactions between the transmembrane segments were analyzed. All polar interactions between the TMs in the CXCR3/GP complex simulation are listed in Table S12. Unexpectedly, the transmembrane segments remained in constant movement (Figure S5) compared to when a chemokine was present, where only some of the TMs had relevant rotations. Additionally, a constant interaction between TM3, TM4, TM5 and TM6 with TM7 was present through the interactions of residues A127-S304, L130-Y308, and N134-Y308 between TM3 and TM7, D186-K300 between TM4 and TM7, Q219-K300 between TM5 and TM7 in the first 400 ns, and Y271-K300 between TM6 and TM7. These results may suggest that the CXCR3 receptor without stimulation of any of the chemokines is in constant movement, and when the chemokine is present, these movements are diminished, stabilizing the TM rotations towards a meta-active conformation. The TM7 transmembrane segment could act as a central axis of an inactive state, since the main interactions with TM3, TM4, TM5 and TM6 with Y308 and K300 are between relevant residues for the stabilization conformations of the meta-active and active states of the receptor as the N132-D186-Q219 interaction network.

To assess if these conformational changes promote the second activation step of CXCR3, a 250-ns AA-MD simulation was performed, taking cluster1 of the CXCR3-CXCL10 CG-MD simulation as the starting structure.

#### 2.4.2. Second Activation Step of the CXCR3-CXCL10 Complex

Chemokine CXCL10 is relevant in vitiligo due to an increased concentration in the serum and skin of patients. Thus, this chemokine is an important marker to assess the progression of vitiligo. To verify the second activation step of CXCR3, a 250-ns simulation was performed, preserving the disulfide bridges and adding the post-translational modifications present in CXCR3. Using cluster1 of the CG-MD simulation at 787.9 ns, which was converted to the all-atom system with the Martini protocol, as a starting structure, which is a state before the active conformation of CXCR3, it represents the interaction between D148 and R149, forming the arginine cage, and G_i/0_ was added in the complex with GTP and Mg^+2^. The conformations present every 25 ns were analyzed, with the conformation at 0 ns obtained after the minimization and equilibrium processes of the system taken as a reference.

**Figure 19:**
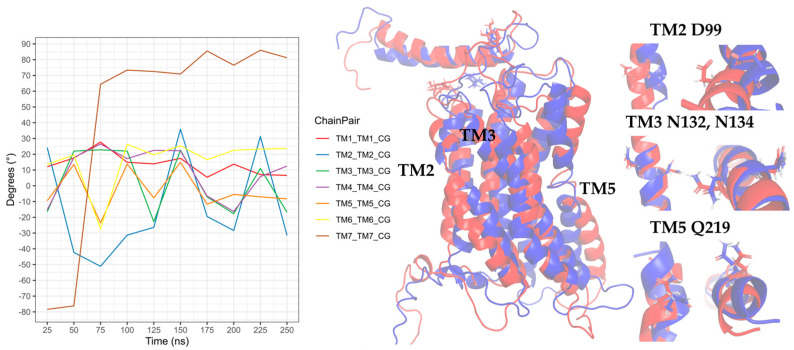
Rotation of TMs from the 250-ns AA-MD simulation of CXCR3-CXCL10-G_i/0_. Rotations of the TMs throughout the simulation are presented, with the relevant rotations of TM1, TM2, TM3, TM5, and TM6. TM7 has an intense rotation at 50 ns. In blue, the initial conformation; in red is the conformation at 600 ns.

The rotation of TM5 and TM6 was observed after 50 ns, and there was a change in the rotation at 175 ns, where the TMs converged, with a greater intensity of TM2, TM3, and TM5. TM7 showed an intense rotation at 50 ns, after maintaining a behavior with discrete rotations that coincided with the rotation of TM2, TM3, and TM5 at 175 ns, as seen in Figure 19. Compared with the rotation in the CG-MD simulation, the transmembrane segments presented a discrete rotation, although with similar behaviors of TM2, TM3, TM4, TM5, and TM6. The rotation of TM2 was necessary for the active conformation of the receptor.

The polar interactions between CXCL10 and CXCR3 decreased compared to the 0 ns between the different time frames analyzed; however, the hydrophobic interactions increased as the simulation progressed. Post-translational modifications are relevant for the interaction of CXCL10 with CXCR3, and the interaction between glycosylation in N32 and K87 of CXCL10 occurred at 25 ns. Additionally, interactions with phosphorylation in Y27 and K87-K88 occurred from 50 ns to 175 ns (Table S12).

The interactions of the transmembrane segments changed in the presence of a functional GP. The most relevant were the loss of the interaction of TM3-TM5 between N132 and Q219, which may be due to the influence of G_i/0_. The interactions of TM3-TM4 between N132 and D186 were maintained at different points of the simulation; at the same time, an interaction between TM3 and TM6 was formed through N134-W268. From 50 to 150 ns, interactions of TM5 and TM6 between R216 and Q219 with Y271 were present. These changes promoted the stabilization of the CXCR3-CXCL10-G_i/0_ complex in the first 100 ns of the simulation (Table S14).

**Figure 20:**
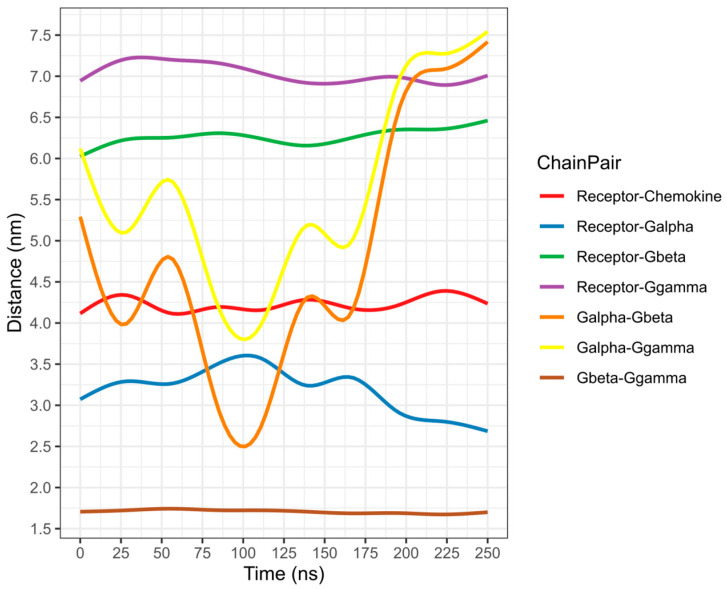
The distance of complex proteins in the AA-MD 250-ns simulation of CXCR3-CXCL10. The separation of the βγ complex to the α subunit occurs at 175 ns.

Once the conformation of CXCR3 and GP stabilized, separation of the βγ complex from the α_i/0_ subunit of GP began at 175 ns, coinciding with the constant changes in the rotation of the transmembrane segments until the end of the simulation (Figure 20). Moreover, the presence of the N132-D186 and N134-W268 interactions and the interaction between R149 of CXCR3 with R193 of the alpha subunit was present in the simulation. Additionally, the interaction between N132 and D186 and N134 and W268 stabilized the active conformation of CXCR3 and promoted the interaction between R149 of CXCR3 with R193 of the α subunit necessary for GP activation (Table S15). This result confirmed that CXCR3 presented a meta-active state with the orientation of CXCL10 towards the extracellular loops and the rotation of the transmembrane segments and, when coupled with the functional G_i/0_, promoted the conformational changes in the α subunit that were promoted for the separation of the βγ-complex, stabilizing the active state through the interactions between N132-D186 and N134-W268. Additionally, it was reported that the N132 residue is fundamental for activity and chemokine binding. When this residue is mutated, the expression in the membrane and activity is diminished [60].

### 2.5. General Protein–Ligand Interaction Model of CXCR3 Antagonists

To provide information to support the design of new antagonists to CXCR3, a general protein–ligand interaction model was constructed based on 894 ligands reported as antagonists in the ZINC public database. This interaction model is similar to a pharmacophore, which is a representation of the chemical features necessary for the recognition of a ligand by macromolecules; in this case, the general protein–ligand interaction model provides these chemical features [61]. Three different conformations of CXCR3 were used—the initial structure (F0) of CXCR3-CXCL10_CG-MD, the most representative conformation (C1) of CXCR3-CXCL10_CG-MD, and the conformation at 175 ns (F7) of CXCR3-CXCL10_AA-MD—to observe if the antagonists could bind before and after the two receptor activation steps occurred; thus, CXCL10 was removed from each complex. Once the molecular docking complexes were obtained, the interactions present in the complexes were grouped for the construction of the general protein–ligand interaction model to observe the chemical features and three-dimensional position, which are most relevant for the interaction of antagonists with the three conformations of CXCR3. Three general protein–ligand interaction models were built, one for each CXCR3 conformation.

#### 2.5.1. General Protein–Ligand Interaction Model of Conformation F0 of CXCR3-CXCL10_CG-MD

**Figure 21:**
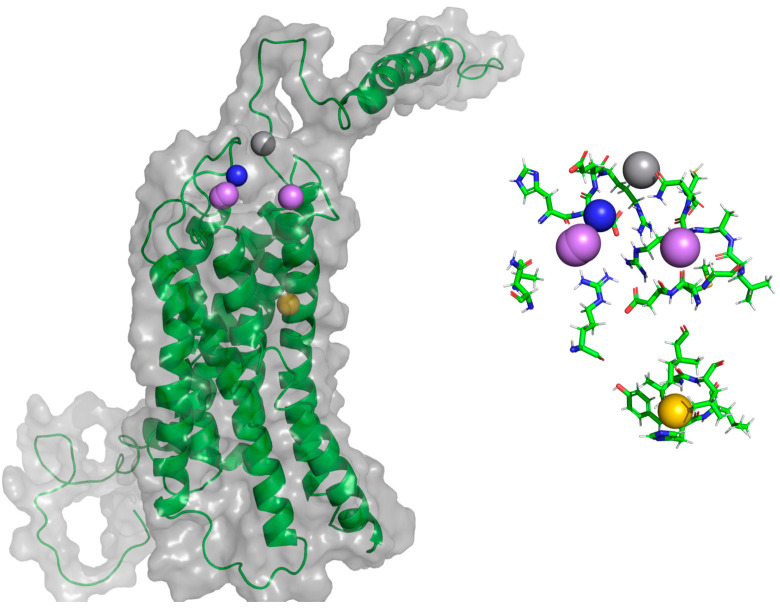
General protein–ligand interaction model of F0 CXCR3-CXCL10_CG-MD. In magenta: hydrogen bond acceptors (HAc), gray: hydrogen bond donors (HDn), yellow: hydrophobic groups (Hph), and blue: positive-charged groups (Pin).

In this structure, CXCR3 presents an inactive conformation and CXCL10 interacts through the first residues of the extracellular region of CXCR3. The position of the hydrogen bond acceptors (HAc), hydrogen bond donors (HDn), and positive-charged (Pin)-type pharmacophoric elements in the region of the extracellular loops, and a hydrophobic (Hph)-type pharmacophoric element between TM5 and TM6, are shown in Figure 21. Residues around 5 Å for each pharmacophoric element are relevant for the interaction and antagonistic activity of the ligands with CXCR3 (Table 5).

The residues R197 and R212 are found around the pharmacophoric elements and are relevant for the binding and activity of all chemokines; D282 is relevant for the binding and activity of CXCL9 and CXCL10. Moreover, residues around the CWTP sequence, a conserved sequence in the GPCRs of class A (CWxP), which are residues related to the rotation of TM6 [40] necessary for CXCR3 activation, are close to the Hph-type pharmacophoric element. This general protein–ligand interaction model, calculated from antagonists docking to an inactive CXCR3 conformation, suggest that these antagonists could hinder the interactions among the chemokines and the residues of the extracellular loops and, subsequently, inhibit the rotation of TM6.

#### 2.5.2. General Protein–Ligand Interaction Model of Conformation C1 CXCR3-CXCL10_CG-MD

**Figure 22:**
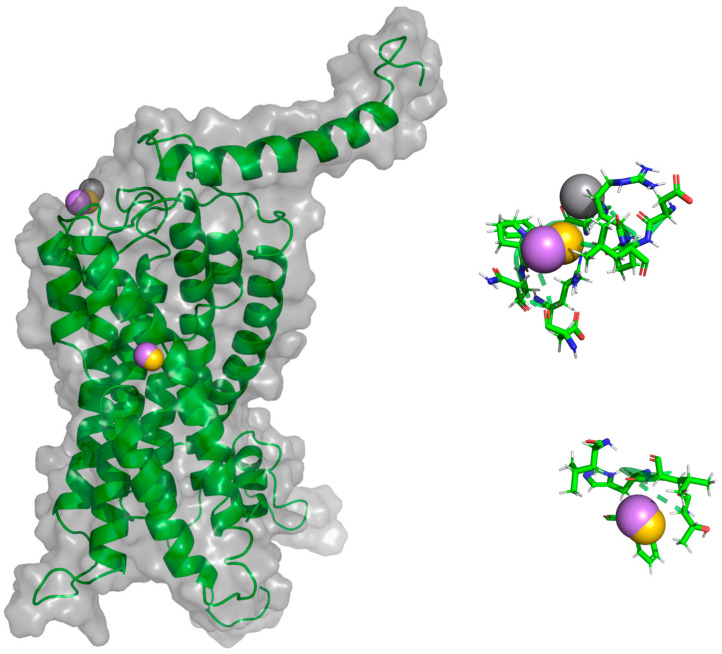
General protein–ligand interaction model of C1 CXCR3-CXCL10_CG-MD. In magenta: HAc, gray: HDn, yellow: Hph, and blue: PIn.

In this structure, CXCR3 is in a pre-active conformation where the first step of activation of the receptors to the chemokines has already occurred. Like the F0 general protein–ligand interaction model, pharmacophoric elements are concentrated in the extracellular loops and the region between TM5 and TM6 (Figure 22). The Hph-type pharmacophoric element is positioned close to residue T269, the residue second to W268 in importance for TM6 rotation; similarly, residues relevant for CXCR3 activation, such as R197 and R212 (Table 6), are found around the elements of HAc, HDn, and Hph.

#### 2.5.3. General Protein–Ligand Interaction Model of Conformation F7 CXCR3-CXCL10_AA-MD

**Figure 23:**
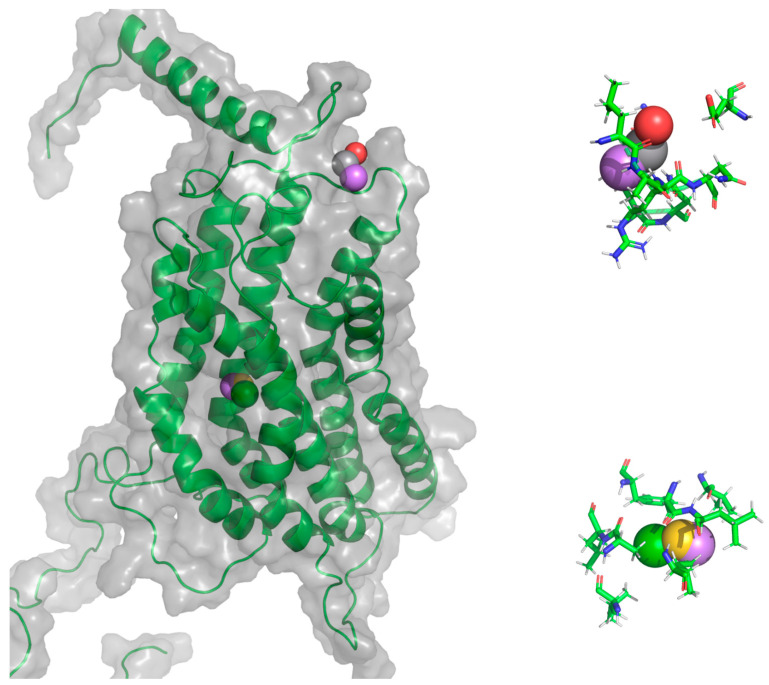
General protein–ligand interaction model of F7 CXCR3-CXCL10_CG-MD. In magenta: HAc, gray: HDn, yellow: Hph, green: aromatic rings (Arm), and red: negative-charged groups (Nin).

This structure corresponds to the active state of the receptor once the chemokine is oriented towards the TMs, causing the conformational changes in the TMs for the coupling of the GP and the subsequent separation of the βγ subunit. In this conformation, the general protein–ligand interaction model can be divided into two: first, the pharmacophoric elements are positioned in the region of the extracellular loops, and second, the pharmacophoric elements are in TM2, TM3, and TM4. Additionally, the latter presented an aromatic rings (Arm) and negative-charged (Nin)-type pharmacophoric element that was absent in the previous general interaction models (Figure 23).

The residues around the pharmacophoric elements were different from the residues of the previous general interaction models. However, the relevant residues for the activation of CXCR3 are listed in Table 7, such as L198 and N199, residues close to R197, which is essential for the binding and activity of chemokines. Thus, antagonist binding in the zone between TM2, TM3, and TM4, shown by the calculated general protein–ligand interaction model, could be related to the destabilization of the interactions of TM3 with the α subunit of GP.

Comparing the three general interaction models, the pharmacophoric elements HAc, HDn, and Hph are the most relevant for the interaction and activity with CXCR3. The three general protein–ligand interaction models differ to some extent, principally between F0 and F175 ns, suggesting that the conformation that occurs in CXCR3 is important in antagonist binding. The general interaction models of F0 and C1 are more relevant, because they can avoid the coupling of the GP, stopping the changes in the first activation step of CXCR3 and inhibiting the chemotaxis derived by receptor stimulation.

Finally, since the antagonists used for general interaction model building were obtained mostly through the HTS campaigns and subsequent optimization [42,43,44,45,46,47,48,49,50,51], the library had considerable structural diversity, with at least eight different structural scaffolds. Therefore, although the calculated general interaction models consisted of 6–8 pharmacophoric elements, they provided information on the important characteristics of the compounds, regardless of the structural scaffold, in addition to the type of major interactions and the residues relevant to the binding of the compounds. All of the above could help in the future rational designs of CXCR3 antagonists.

## 3. Materials and Methods

### 3.1. Protein Structures and Homology Modeling

The primary sequence of the full legth-CXCR3A receptor, the chemokine CXCL9, and the α_i/0_ subunit of the G-protein were obtained from the UniProt database [58] (codes P49682, Q07325, and P04899, respectively). The three-dimensional structures of CXCL10, CXCL11, and the G-protein βγ subunits complex were obtained from the Protein Data Bank (PDB) database with codes 1LV9 [62], 1RJT [32], and 6G79 [63], respectively. The amino acid sequences of CXCR3, CXCL9, and Gα_i/0_ were uploaded to the GPCR-I-TASSER server for CXCR3 and the general module I-TASSER server for CXCL9 and Gα_i/0_ [64] for homology model building. The CXCR3 and CXCL9 models were calculated with the best sequence identity found by the BLAST server (CCR5 and CXCL2, respectively) [65,66].

### 3.2. Protein-Protein Docking

To build the CXCR3-chemokine complex, molecular docking was performed using the ClusPro server [67,68] with the structures from the PDB codes stated above for CXCL10 and CXCL11 and the most representative conformation of the molecular dynamics simulations (cluster1) for CXCR3 and CXCL9. Relevant residues that were reported for the interaction of chemokines with the receptor and their subsequent activation are shown in Table 8. Therefore, the proximity to the first 16 residues and Y27 and Y29 of CXCR3 was considered as a restriction for molecular docking. In the case of the chemokines, no restriction was used [59,69,70].

### 3.3. Molecular Dynamics (MD) Simulations

The preparation of all the systems for MD simulations, including the addition of post-translational modifications (disulfide bridges, glycosylations, and lipidations) present in the proteins, was undertaken on the CHARMM-GUI web server [71,72]. In the case of sulfation in CXCR3, phosphorylation was used to generate a similar electrostatic environment to the original, because the parameters of sulfation are not readily available in CHARMM-GUI [71,72,73]. MD simulations were performed with Gromacs 2018.7 [74,75] using an isothermic-isobaric ensemble (NPT) at 1 atm and 310.15 K, employing a temperature coupling and velocity rescaling with a stochastic term [76] and a Parrinello-Rahman barostat [77]. The systems were submitted to 5000 steps steepest-descent minimization, followed by the 6-equilibration steps scheme by reducing the force constants suggested by the CHARMM-GUI server prior to performing the production simulations. The TIP3 water model and POPC lipids were used. The modules “distance” and “cluster” of Gromacs 2018.7 were used to measure the distance between each chemokine and CXCR3 and to find the relevant conformations of the simulation using the “gromos” algorithm [78], respectively. All graphs were built with R [79] using ggplot2 libraries [80].

All the MD simulations were performed in the ADA cluster of the National Laboratory of Advanced Scientific Visualization at Campus Juriquilla of the National Autonomous University of Mexico (LAVIS-UNAM).

#### 3.3.1. All-Atom Molecular Dynamics (AA-MD) Simulations

##### Relaxation of the Homology Models

Once the homology models were calculated, all systems for the molecular dynamics simulations of CXCR3 were built using the Membrane Builder module in CHARMM-GUI; CXCL9 was built using the Solution Builder module in CHARMM-GUI. In the all-atom systems, the CHARMM36 force field was used in an NPT ensemble, as stated previously. The production simulations for model relaxation were 50-ns-long for CXCR3 and 5 ns for CXCL9.

##### Relaxation of the Chemokine-CXCR3/GP Complexes

Once the homology models were relaxed, the G_i/0_ protein was added to the conformation representative of CXCR3 simulation, and a 100-ns molecular dynamics simulation system was produced for relaxation of the CXCR3/GP complex. Next, the system was prepared for the CXCR3-chemokine-GP complexes with a 50-ns simulation to observe the evolution of the system. Interactions between CXCR3 and the chemokines were observed by LigPlot+ [81,82] and the ESBRI Server for salt bridges [83,84,85] using representative structures from the simulations. The RMSD of this simulation is shown in Figure S6.

#### 3.3.2. Coarse-Grained Molecular Dynamics (CG-MD) Simulations

To assess if the receptor reached an activated conformation, the systems for the molecular dynamics simulations of CXCR3/chemokines were built using the Martini Maker [86] module in CHARMM-GUI; the Martini2.2p force field was used in an NPT assemble at 1 atm and 310.15 K, and the coarse-grained simulations of 1 μs were produced. Representative structures of each simulation were analyzed in LigPlot+ and ESBRI to observe the interactions between CXCR3 and the chemokines.

Finally, a 250-ns AA-MD simulation was performed; the system using the representative structure of the biggest conformation cluster of CXCR3 and CXCL10 (cluster1) of the CG simulation was built, and the GP in the complex with guanosine triphosphate (GTP) and Mg^2+^ was added. This system was built as previously described for the AA-MD simulations. The RMSD of these simulations are shown in Figures S6 and S7.

### 3.4. Ligand Docking and the General Protein–Ligand Interaction Model

#### 3.4.1. Ligand Docking

The 3D structures of 894 unique structures of reported antagonists of CXCR3 [42,43,44,45,46,47,48,49,50,51] were obtained from the ZINC public database [87], with pKi values (minus logarithm of the equilibrium dissociation constant of an antagonist) [88] ranging from 5 to 9.7. A grid of 40 × 32 × 40 Å with spacing of 0.375 Å, centered on the residues identified as relevant for both receptor activation and agonist binding, was calculated with AutoGrid 4.2.6 [89]. Finally, molecular docking was performed with AutoDock 4.2.6 optimized for graphics-processing units, using a total of 25 runs and 25,000,000 evaluations, with a Lamarckian genetic algorithm and Solis–Wets local search [90] using three different conformations of CXCR3: the initial frame (F0) of the CXCR3-CXCL10 complex coarse-grained molecular dynamics simulation (CXCR3-CXCL10_CG-MD), the most representative conformation (C1) of the CXCR3-CXCL10_CG-MD simulation, and frame 7 (F7) corresponding to the 175-ns timestep of the 250-ns all-atom molecular dynamics (AA-MD) simulation of CXCR3-CXCL10.

#### 3.4.2. Pharmacophoric Elements Identification and General Protein–Ligand Interaction Model Calculation

The software Pharmer (2015, Pittsburg, PA, USA) [91] was used to identify the key pharmacophoric elements of the docked ligands in their most frequent binding conformations, obtained by clustering the docking results of each ligand with a root mean standard deviation (RMSD) cutoff of 2 Å on their protein–ligand complexes: hydrogen bond acceptors (HAc), hydrogen bond donors (HDn), aromatic rings (Arm), hydrophobic groups (Hph), positive-charged groups (PIn), and negative-charged groups (NIn) in the three-dimensional conformations of the docked ligands. These characteristics were clustered according to their nature, position, and size using a partition-around-medoids statistical method [92], implemented in the cluster package [93], available in the statistical software R (3.5.2, Vienna, Austria) [79].

### 3.5. Manipulation of Complexes and Figures

All of the protein figures presented in this article and the alignments were made in the PyMOL program [94]. The manipulation of the protein complexes was performed using the academic version of the Schrödinger-Maestro program [95].

## 4. Conclusions

A homology model of CXCR3 was calculated to build the CXCR3/Gα_i/0βγ_ complex and study the interactions with its chemokines through AA-MD and CG-MD simulations. The early interaction of CXCL9 with a glycosylation of the receptor was observed, which indicates the importance in the first interactions of CXCL9 with CXCR3. These interactions enable the conformational change of the system towards a favorable state for hydrogen bonds and hydrophobic and salt bridge interactions. CG-MD simulations showed the first activation step of CXCR3, which corresponds to the binding and orientation of the chemokines towards the transmembrane region and the conformational change of the receptor towards a functional state for the coupling of the GP. Later, the 250-ns AA-MD simulation confirmed the importance of post-translational modifications for the binding and activity of CXCL10, showing interactions with phosphorylation in Y27 throughout the simulation, and glycosylation in N32 occurred in the first 25 ns, indicating that glycosylations form a relevant interaction between the chemokine and receptor. Next the changes in the rotations of the transmembrane segments, the interaction between R149 and D193 of the α subunit, and the separation of the βγ complex confirmed the second activation step. Thus, a simulation of the process by which CXCR3 is activated by CXCL10 was obtained, in which N132-D186 and N134-W268 were the relevant interactions to stabilize the active state of the receptor in the presence of GP complexed with GTP and Mg^+2^. Moreover, the pharmacophoric elements HAc, HDn, Hph, and PIn are the most relevant for the binding and activity of the antagonists listed in the ZINC database: most of those are in two regions of the receptor: the extracellular loops and the TM5-TM6 region. Antagonist binding in the first region could prevent the orientation of the chemokine towards the TMs and the interaction of the chemokine with the extracellular loops, and binding in the second region could hinder the rotation of TM6 to activate the receptor. These general protein–ligand interaction models can be used to design new molecules to inhibit the first step of activation in CXCR3. Furthermore, these results allow a better understanding of the activation of two steps of CXCR3, the involved residues, and the rotation of transmembrane segments, in agreement with the reported experimental data. This information is relevant for the design of molecules that can prevent the binding of chemokines to CXCR3, the orientation towards the transmembrane region, and the activation of GP. As a consequence of antagonizing CXCR3, a reduction of the chemotaxis of the cells of the immune system could prevent the appearance of new lesions and the progression of existing lesions in vitiligo patients. Additionally, the antagonism of CXCR3 can inhibit other pathological processes in which CXCR3 is involved, such as cancer metastasis. In contrast, CXCR3B is a receptor with 100 residues in the N-terminus, compared to the 53 residues on CXCR3A, and is related with melanocytic apoptosis in the early stages of vitiligo; therefore, the general protein–ligand interaction model reported here could be relevant also in CXCR3B, a question that stands as a perspective for future works. Still, it is particularly important that these results be experimentally validated in collaboration with experts in this area.

## Figures and Tables

**Table 1:** Timestep and clustering of the AA-MD simulations.

	Most Representative Conformation Timestep (ns)				
Complex	5 ns	50 ns	100 ns	Cutoff (nm)	Clusters
CXCR3		32.1	86.9	0.4 */0.2 **	3 */10 **
CXCL9	3.8			0.2	14
CXCR3-CXCL9		31.1		0.4	9
CXCR3-CXCL10		33.3		0.51	3
CXCR3-CXCL11		26.6		0.4	10

* 50-ns simulation and ** 100-ns simulation. AA-MD: all-atom molecular dynamics.

**Table 2:** Timestep and clustering of the coarse-grained molecular dynamics (CG-MD) simulations.

	Most Representative Conformation Timestep (ns)				
Complex	Cluster1	Cluster2	Cluster3	Cutoff (nm)	Clusters
CXCR3-CXCL9	607.0	131.0	39.0	0.6	10
CXCR3-CXCL10	787.9	159.6	51.2	0.6	10
CXCR3-CXCL11	532.7	171.3	979.3	0.6	10

**Table 3:** Presence of the so-called arginine cage in the CG-MD simulation.

Time Frame (ns)	CXCR3_CXCL9	CXCR3_CXCL10	CXCR3_CXCL11
0	X	X	X
100	X		X
200	X		X
300	X		X
400	X		
500	X		
600			
700	X	X	
800			X
900			
1000	X		

**Table 4:** Transmembrane segments in CXCR3.

TM1	TM2	TM3 *	TM4	TM5 *	TM6	TM7
54–80	90–110	126–147	170–189	213–233	256–277	299–321

* Some residues were included in the analysis; in TM3, the residues 148–150 are the DRY motif, and the residue 234 and 235 are relevant in the TM5 rotation.

**Table 5:** Pharmacophoric elements of F0 CXCR3-CXCL10_CG-MD.

Pharmacophoric Element	Residues (5 Å)
HAc	A192, **H194**, **D195**, R212, M281, D282, G284, L286, A287, **R288**
HDn	**D195**, **E196**, **R197**, N289, C290
Hph	Y271, H272, L273, V275, L276, I279
PIn	**H194**, **D195**, **E196**, **R197**, **R288**

Bold is used to show the residues that are repeated in the different pharmacophoric elements. HAc: hydrogen bond acceptors, HDn: hydrogen bond donors, Hph: hydrophobic groups, and Pin: positive-charged groups.

**Table 6:** Pharmacophoric elements of C1 CXCR3-CXCL10_CG-MD.

Pharmacophoric Element	Residues (5 Å)
HAc	**R197**, **L198**, **P208**, **Q209**, **F224**, **T269**, **H272**, **L273**, L276, D282
HDn	D195, **E196**, **R197**, N206, F207, **R212**
Hph	**E196**, **R197**, **L198**, **P208**, **Q209**, **R212**, **F224**, **T269**, **H272**, **L273**

Bold is used to show the residues that are repeated in the different pharmacophoric elements.

**Table 7:** Pharmacophoric elements of F7 CXCR3-CXCL10_AA-MD.

Pharmacophoric Element	Residues (5 Å)
Arm	**A129**, **I133**, L180, **A183**, L184, **F187**
HAc	**V126**, **A129**, **L130**, **I133**, A183, **F187**, **L198**, **N199**, **L286**, A287, **R288**
HDn	S36, **L198**, **N199**, **L286**, **R288**, N289
Hph	**V136**, **A129**, **L130**, **I133**, **A183**, **F187**
NIn	E31, **L286**, A287, **R288**

Bold is used to show the residues that are repeated in the different pharmacophoric elements. Arm: aromatic rings and Nin: negative-charged groups.

**Table 8:** Relevant amino acids for the interactions between CXCR3 and its chemokines.

Residues	Observation
Residues 1–16	Relevant for interactions between the CXCR3 receptor and its chemokines [28].
R216	Fundamental for chemotaxis and the mobilization of Ca^+2^ but not in the union of chemokines [59].
Y27, Y28	Sulfation in these residues is necessary for the interactions of the chemokines [28,31,59].
N197, N212	Essential for the union of CXCL10 and CXCL11 [59].
D112, D278	Relevant in the interactions of the three chemokines, but only CXL10 and CXCL11 have an influence on the activity [59].
D282, E293	Influence on the activity of CXCL9 and CXCL10 and the interaction of CXCL11 [59].

## Supplementary Materials

The following are available online. Figure S1: Ramachandran plot. Figure S2: Ramachandran plot. Figure S3: Ramachandran plot. Figure S4: AA-MD simulation of CXCL9 5 ns. Figure S5: Rotation of the TMs from the CG-MD simulation of the CXCR3/GP complex. Figure S6: RMSD of the AA-MD simulations. Figure S7: RMSD of the CG-MD simulations. Table S1: Salt bridge interactions of CXCR3 with CXCL9, CXCL10, and CXCL11 in molecular docking. Table S2: Salt bridge interactions of CXCR3 with CXCL9, CXCL10, and CXCL11 in AA-MD 50 ns. Table S3: Salt bridge interactions of CXCR3 with CXCL9 in the CG-MD simulation. Table S4: Salt bridge interactions of CXCR3 with CXCL10 in the CG-MD simulation. Table S5: Salt bridge interactions of CXCR3 with CXCL11 in the CG-MD simulation. Table S6: Polar interactions between CXCR3-CXCL9 CG-MD. Table S7: Polar interactions between the TMs in CXCR3-CXCL9 CG-MD. Table S8: Polar interactions between CXCR3-CXCL10 CG-MD. Table S9: Polar interactions between the TMs in CXCR3-CXCL10 CG-MD. Table S10: Polar interactions between CXCR3-CXCL11 CG-MD. Table S11: Polar interactions between the TMs in CXCR3-CXCL11 CG-MD. Table S12: Polar interactions between the TMs in the CXCR3/GP complex 1μ. Table S13: Polar interactions between CXCR3-CXCL10 250-ns AA-MD. Table S14. Polar interactions between the TMs in CXCR3-CXCL10 250-ns AA-MD. Table S15: Polar interactions between CXCR3_alpha subunit 250-ns AA-MD.
