# Supplementary material for: Computational Study of C-X-C Chemokine Receptor (CXCR)3 Binding with Its Natural Agonists Chemokine (C-X-C Motif) Ligand (CXCL)9, 10 and 11 and with Synthetic Antagonists: Insights of Receptor Activation towards Drug Design for Vitiligo
Source: Molecules. 2020 Sep 25;25(19):4413. doi: 10.3390/molecules25194413 (PMC7582348; doi:10.3390/molecules25194413)
Supplement: Supplementary file 1 [file molecules-25-04413-s001.pdf]

Supplementary information.

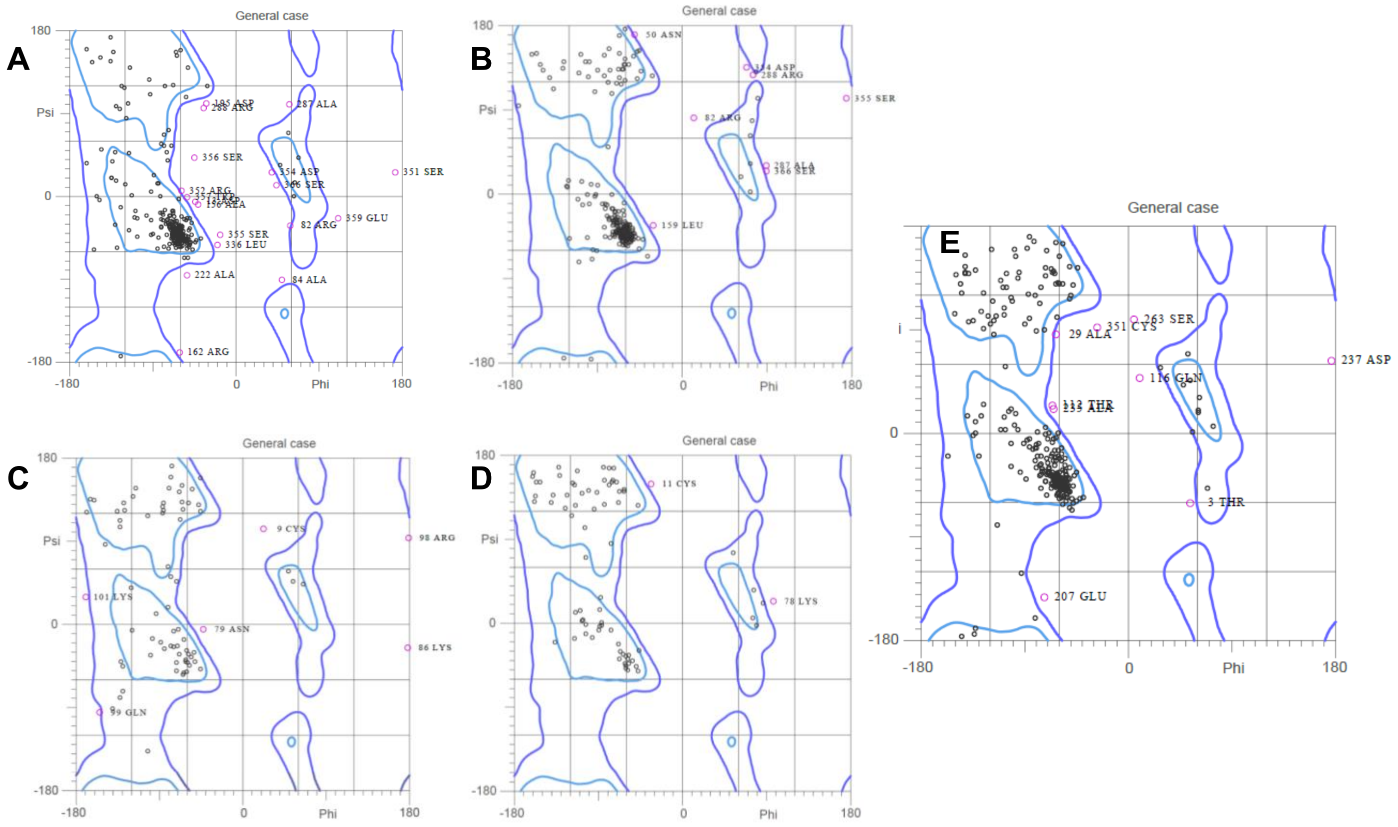

Figure S1. Ramachandran Plot. A) CXCR3_model_I-TASSER, B) CXCR3_Cluster_1_50 ns, C) CXCL9_model_I-TASSER, D) CXCL9_Cluster_1_5 ns, and E) Alpha_model_I-TASSER

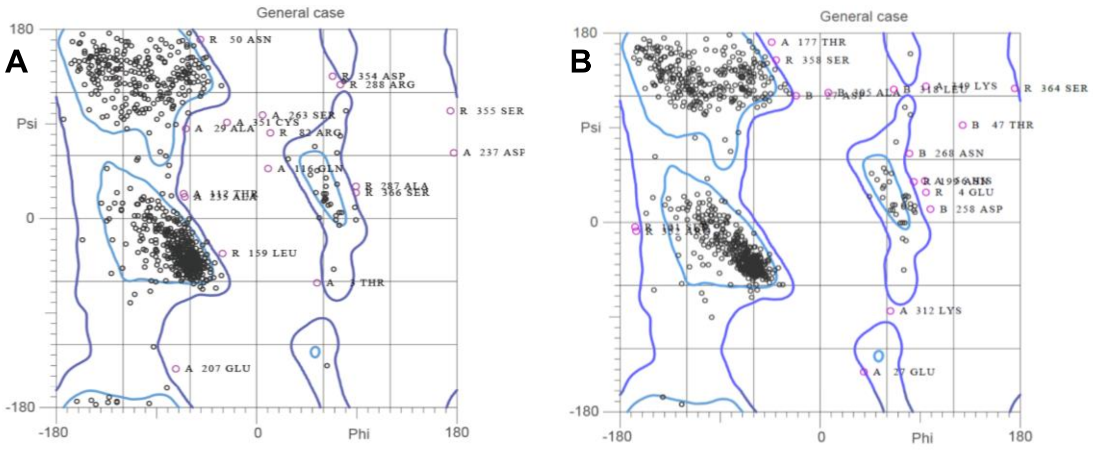

Figure S2. Ramachandran Plot. A) CXCR3/$G_{\alpha i/0\beta\gamma}T_0$ and B) CXCR3/$G_{\alpha i/0\beta\gamma}$Cluster_1_100 ns.

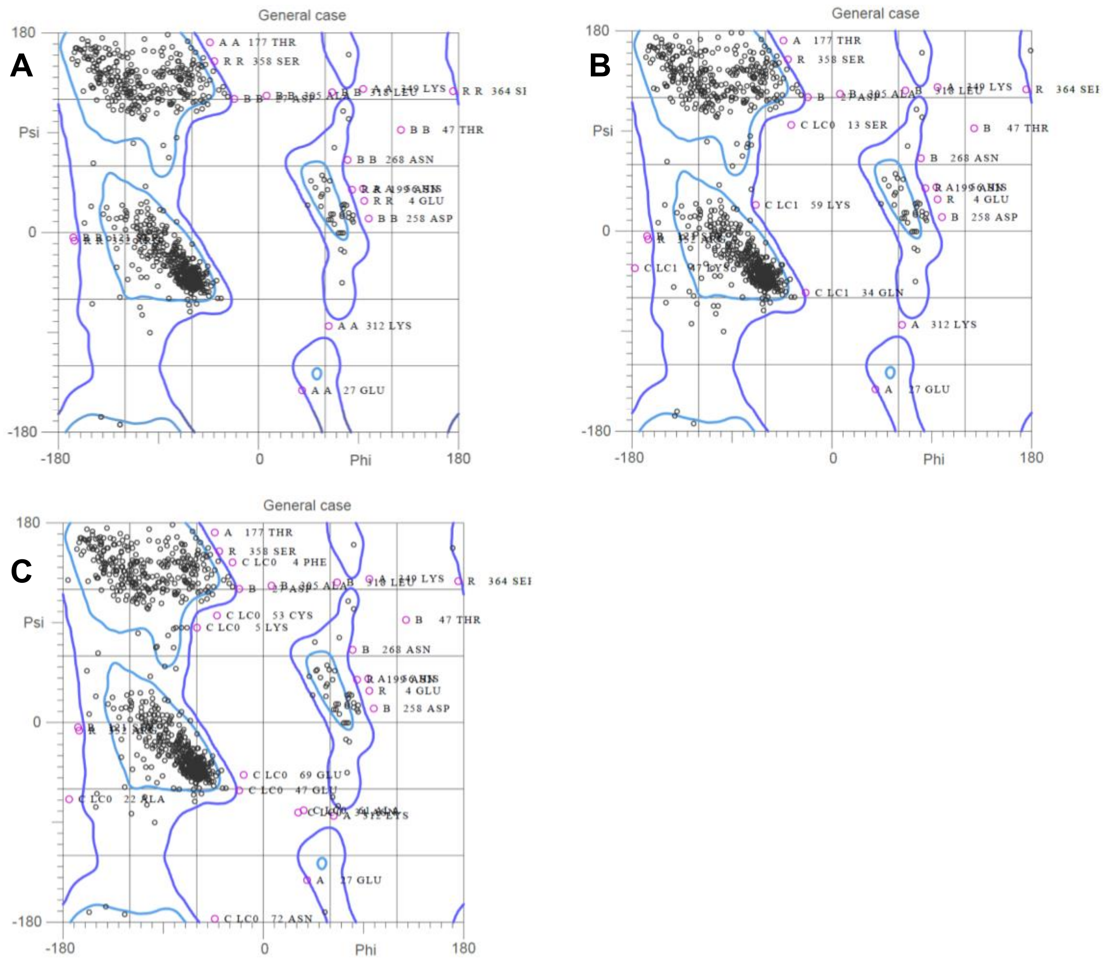

Figure S3. Ramachandran Plot. A) CXCR3_CXCL9_Docking, B) CXCR3_CXCL10_Docking, C) CXCR3_CXCL11_Docking.

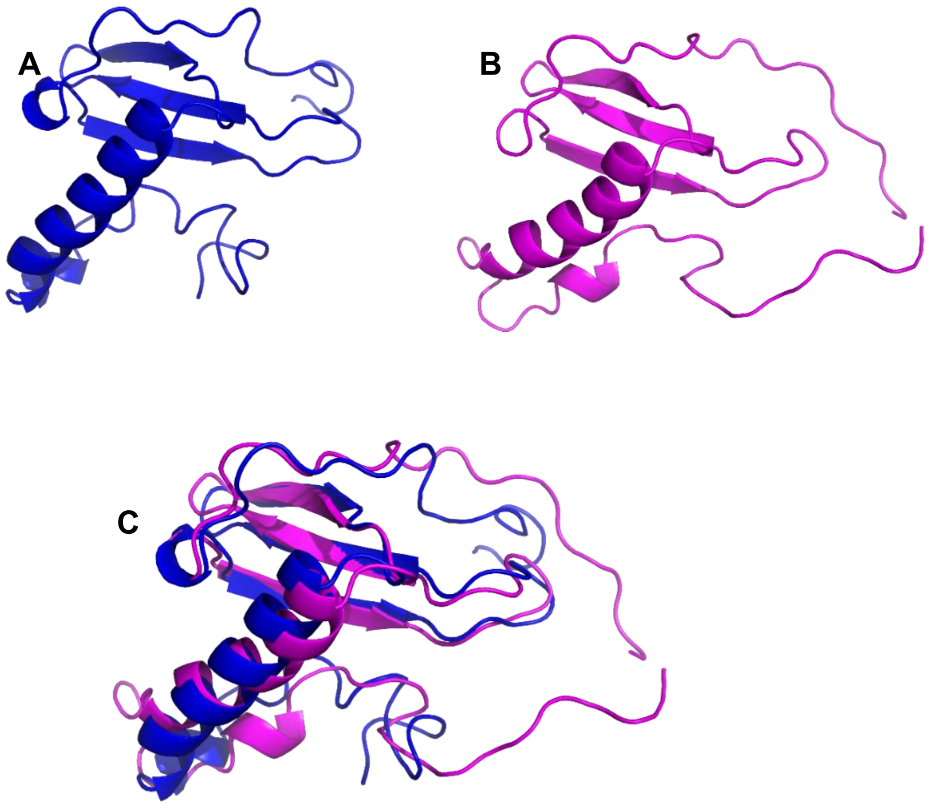

Figure S4. AA-MD simulation of CXCL9 5 ns. A) CXCR3 model obtained from **I-**TASSER, B) Cluster_1 of the simulation, C) Alignment of T_0 and Cluster_1, RMSD =2.675 Å.

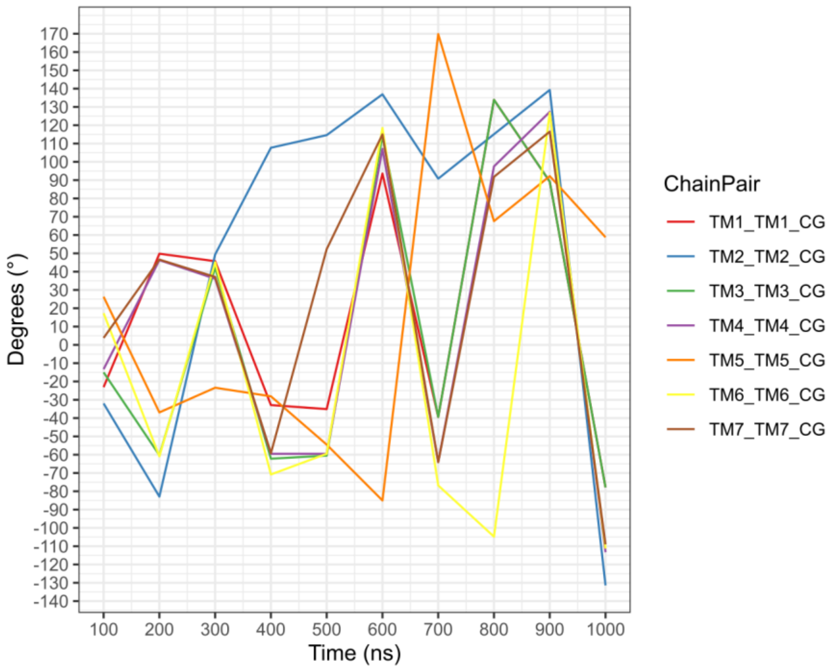

Figure S5. Rotation of TMs from the CG-MD simulation of CXCR3/GP complex.

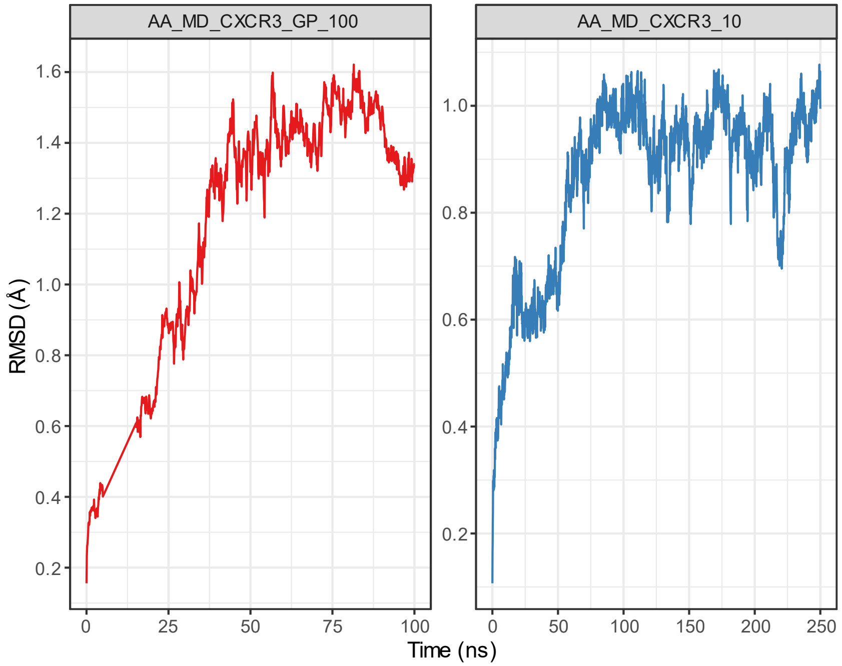

Figure S6. RMSD of AA-MD simulations.

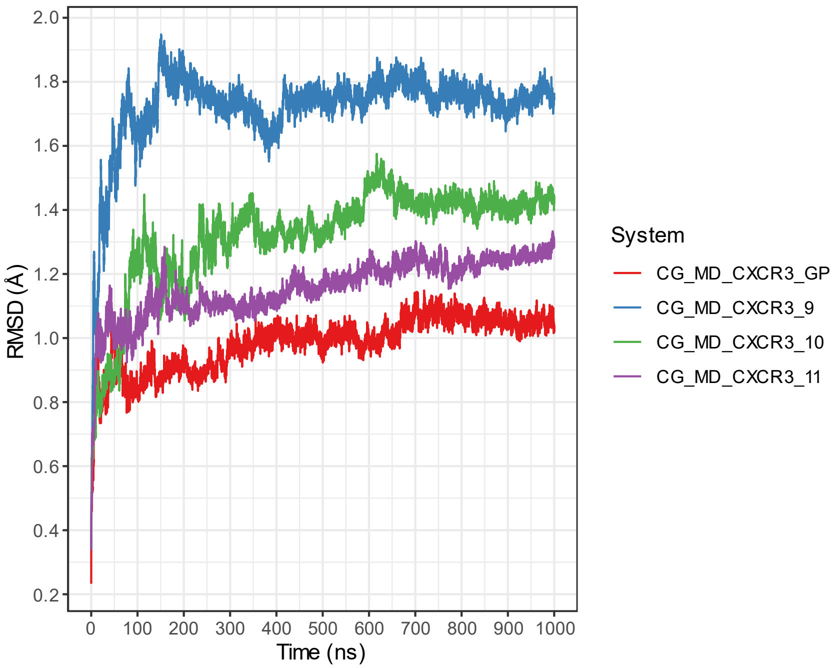

Figure S7. RMSD of CG-MD simulations.

Table S1. Salt bridge interactions CXCR3 with CXCL9, CXCL10 and CXCL11 in molecular docking. There are two salt bridges between CXCR3 and CXCL9, one between CXCR3_CXCL10, and none between CXCR3 and CXCL11.

CXCL9	CXCR3	Distance
NH1 ARG Q 30	OD1 ASP R 7	3.15
NH1 ARG Q 30	OD2 ASP R 7	2.88

CXL10	CXCR3	Distance
NH1 ARG Q 29	OE1 GLU R 15	3.79

Table S2. Salt bridge interactions CXCR3 with CXCL9, CXCL10 and CXCL11 in AA-MD 50 ns. There are nine salt bridges between CXCR3 and CXCL9, none between CXCR3 and CXCL10, and one between CXCR3_CXCL11.

CXCL9	CXCR3	Distance
NH1 ARG Q 27	OE1 GLU R 4	2.60
NH2 ARG Q 27	OE1 GLU R 4	2.90
NZ LYS Q 28	OD1 ASP R 13	2.88
NZ LYS Q 28	OD2 ASP R 13	2.75
NZ LYS Q 47	OE1 GLU R 21	2.76
NZ LYS Q 50	OE1 GLU R 21	3.82
NZ LYS Q 104	OD1 ASP R 28	2.59
NZ LYS Q 104	OD2 ASP R 28	3.71
NZ LYS Q 104	OE1 GLU R 31	2.65

CXCL11	CXCR3	Distance
NZ LYS Q 38	OE1 GLU R 4	3.16
NZ LYS Q 67	OE1 GLU R 4	2.65

Table S3. Salt bridge interactions CXCR3 with CXCL9 in CG-MD simulation. The salt bridges present in the three most representative clusters of the 1µs simulation.

Cluster_1		
**CXCL9**	**CXCR3**	**Distance**
NH1 ARG Q 27	OE1 GLU R 4	2.55
NH2 ARG Q 27	OE1 GLU R 4	3.03
NZ LYS Q 28	OE1 GLU R 15	2.54
NH2 ARG Q 30	OD1 ASP R 7	2.83
NH2 ARG Q 30	OD2 ASP R 7	2.84
NZ LYS Q 60	OD2 ASP R 13	2.50
NZ LYS Q 109	OE1 GLU R 21	3.09
NZ LYS Q 109	OE1 GLU R 196	2.62
NH2 ARG Q 114	OD1 ASP R 28	2.69
NZ LYS Q 115	OD2 ASP R 28	2.53
NZ LYS Q 115	OE1 GLU R 31	3.29
NH1 ARG Q 118	OD1 ASP R 35	2.75
NH1 ARG Q 118	OD2 ASP R 35	3.30
NH2 ARG Q 118	OD1 ASP R 35	3.73
NH2 ARG Q 118	OD2 ASP R 35	2.63
NZ LYS Q 122	OE1 GLU R 196	3.64

Cluster_2		
**CXCL9**	**CXCR3**	**Distance**
NZ LYS Q 28	OE1 GLU R 15	2.59
NH1 ARG Q 30	OD1 ASP R 7	2.62
NH1 ARG Q 30	OD2 ASP R 7	3.71
NH2 ARG Q 30	OD1 ASP R 7	2.82
NZ LYS Q 50	OE1 GLU R 21	3.11
NZ LYS Q 60	OD1 ASP R 13	3.81
NZ LYS Q 60	OD2 ASP R 13	2.52
NZ LYS Q 68	OE1 GLU R 21	2.66
NZ LYS Q 87	OD2 ASP R 7	2.94
NH2 ARG Q 114	OD2 ASP R 28	2.63
NZ LYS Q 115	OD1 ASP R 28	2.64
NZ LYS Q 115	OE1 GLU R 31	2.95
NH1 ARG Q 118	OD1 ASP R 35	2.54
NH1 ARG Q 118	OD2 ASP R 35	3.94
NH2 ARG Q 118	OE1 GLU R 31	3.03
NZ LYS Q 122	OE1 GLU R 196	3.41

Cluster_3		
**CXCL9**	**CXCR3**	**Distance**
NH1 ARG Q 27	OE1 GLU R 4	2.76
NH1 ARG Q 27	OD2 ASP R 7	2.66
NH2 ARG Q 27	OE1 GLU R 4	2.87
NH1 ARG Q 30	OD1 ASP R 7	2.73
NH1 ARG Q 30	OD2 ASP R 7	3.28
NH2 ARG Q 30	OD1 ASP R 7	3.45
NZ LYS Q 50	OE1 GLU R 21	2.64
NZ LYS Q 60	OD2 ASP R 13	2.62
NH1 ARG Q 114	OD2 ASP R 28	2.65
NZ LYS Q 115	OD1 ASP R 28	2.56
NZ LYS Q 115	OE1 GLU R 31	2.59
NH1 ARG Q 118	OD2 ASP R 35	3.51
NH2 ARG Q 118	OD1 ASP R 35	3.17
NH2 ARG Q 118	OD2 ASP R 35	2.64

Table S4. Salt bridge interactions CXCR3 with CXCL10 in CG-MD simulation. The salt bridges present in the three most representative clusters of the 1μs simulation.

Cluster_1		
**CXCL10**	**CXCR3**	**Distance**
NH1 ARG Q 29	OE1 GLU R 15	3.95
NH2 ARG Q 29	OE1 GLU R 15	2.73
NH2 ARG Q 43	OE1 GLU R 4	2.62
NH1 ARG Q 59	OD1 ASP R 13	2.66
NH2 ARG Q 59	OD1 ASP R 13	3.04
NZ LYS Q 67	OD2 ASP R 28	2.56
NZ LYS Q 72	OD1 ASP R 46	3.00
NZ LYS Q 72	OD2 ASP R 46	2.71
NZ LYS Q 80	OD1 ASP R 7	3.45
NZ LYS Q 80	OD2 ASP R 7	2.55
NZ LYS Q 87	OE1 GLU R 31	2.79
NZ LYS Q 91	OE1 GLU R 21	2.97

Cluster_2		
**CXCL10**	**CXCR3**	**Distance**
NH1 ARG Q 29	OE1 GLU R 15	3.17
NH2 ARG Q 59	OD1 ASP R 13	3.28
NH2 ARG Q 59	OD2 ASP R 13	2.74
NZ LYS Q 80	OD1 ASP R 7	2.53
NZ LYS Q 87	OD1 ASP R 28	2.87
NZ LYS Q 87	OD2 ASP R 28	2.71
NZ LYS Q 91	OE1 GLU R 21	3.13

Clsuter_3		
**CXCL10**	**CXCR3**	**Distance**
NH2 ARG Q 29	OE1 GLU R 15	3.33
NH2 ARG Q 59	OD2 ASP R 13	2.98
NH1 ARG Q 73	OE1 GLU R 4	3.16
NH2 ARG Q 73	OE1 GLU R 4	3.95
NZ LYS Q 80	OD1 ASP R 7	2.84
NZ LYS Q 80	OD2 ASP R 7	2.77
NZ LYS Q 91	OE1 GLU R 21	2.88

Table S5. Salt bridge interactions CXCR3 with CXCL11 in CG-MD simulation. The salt bridges present in the three most representative clusters of the 1μs simulation.

Cluster_1		
CXCL11	CXCR3	Distance
NH1 ARG R 197	OE1 GLU Q 90	3.26
NZ LYS Q 26	OD2 ASP R 7	2.57
NH1 ARG Q 27	OE1 GLU R 4	3.21
NH1 ARG Q 27	OD2 ASP R 13	3.40
NH2 ARG Q 27	OE1 GLU R 4	2.71
NH2 ARG Q 27	OD1 ASP R 7	2.68
NH2 ARG Q 29	OD1 ASP R 13	2.95
NZ LYS Q 70	OE1 GLU R 15	2.56
NZ LYS Q 78	OD2 ASP R 52	2.55
NH1 ARG Q 83	OE1 GLU R 21	2.63
NZ LYS Q 87	OD1 ASP R 46	2.72
NZ LYS Q 87	OD2 ASP R 46	2.84
NZ LYS Q 88	OE1 GLU R 293	2.62
NH1 ARG Q 91	OE1 GLU R 33	3.06
NZ LYS Q 92	OE1 GLU R 31	2.72

Cluster_2		
CXCL11	CXCR3	Distance
NZ LYS Q 26	OD2 ASP R 7	3.44
NH2 ARG Q 27	OE1 GLU R 4	3.31
NH1 ARG Q 29	OD2 ASP R 13	2.80
NH2 ARG Q 29	OD1 ASP R 13	3.79
NH2 ARG Q 29	OD2 ASP R 13	1.83
NZ LYS Q 80	OD1 ASP R 46	3.54
NH1 ARG Q 83	OE1 GLU R 21	3.97
NH2 ARG Q 83	OE1 GLU R 21	2.51
NH2 ARG Q 91	OE1 GLU R 33	2.14

Cluster_3		
CXCL11	CXCR3	Distance
NH2 ARG R 197	OE1 GLU Q 90	2.69
NH1 ARG Q 27	OE1 GLU R 4	2.71
NH2 ARG Q 27	OE1 GLU R 4	3.12
NH1 ARG Q 29	OD1 ASP R 13	2.63
NH1 ARG Q 29	OD2 ASP R 13	3.29
NZ LYS Q 38	OD1 ASP R 7	2.81
NZ LYS Q 38	OD2 ASP R 7	2.75
NZ LYS Q 47	OE1 GLU R 15	3.02
NH1 ARG Q 83	OE1 GLU R 21	2.83
NH2 ARG Q 83	OE1 GLU R 21	3.35
NZ LYS Q 87	OD1 ASP R 46	2.64
NZ LYS Q 87	OD2 ASP R 46	2.68
NZ LYS Q 88	OE1 GLU R 196	3.58
NZ LYS Q 88	OE1 GLU R 293	2.71
NH1 ARG Q 91	OE1 GLU R 33	2.67
NH2 ARG Q 91	OE1 GLU R 33	2.79
NZ LYS Q 92	OE1 GLU R 31	2.72

Table S6. Polar interactions between CXCR3_CXCL9 CG-MD.

0 ns		100 ns		200 ns		300 ns		400 ns		500 ns		600 ns		700 ns		800 ns		900 ns		1000 ns	
R	Q	R	Q	R	Q	R	Q	R	Q	R	Q	R	Q	R	Q	R	Q	R	Q	R	Q
D13	K28,S57	E4	R27	E15	Q72,K28	D7	R30	N22	K68	D13	S57,K60	A285	K28	N22	T40,K68	D7	R30,R27	D7	R30	E4	R27
E4	R27	D7	R30	A14	T73	E4	R27,T23	Q9	K60	A17	A53	N12	V26,Q72	L286	K68	E4	R27	N22	K68	H8	R27
V5	R27	D13	K60,I64	D7	R30	L3	T23	D13	K60,S55	E21	K50,Q51,K109	D7	R30,R27	N12	V26	S6	R30	S26	K68	S6	R30
M1	E59,E62	Q9	R30,E62	E4	R27	N12	K28	E15	T7,55	L20	K109	E4	R30,R27	A14	A65	N12	V26	R292	Q72	D7	R30
D7	R30,E59	S24	P54	D195	K123	G284	K28	L11	Q72	D7	Q72,R30,K28	S26	Q38	E15	K28,T66	E15	K28,T66	E15	T66	E21	K109,Q51
S6	G29	D28	K115,R114	N206	K123	E15	G29	D7	G29,R30	E15	K28,N69	N22	K68	L11	Q72	D13	K60	N12	K28	D112	K123
E21	N69	N32	R114	H194	K123	A14	T66	S6	R30	G284	K28	E15	T66	D7	R30, R27	Q9	K60	Q45	N69	D13	S57,K60
		E31	K115,R118	E196	K122	N22	N69,K68	S25	D48	E4	R27	D13	K60	E21	V113,K109	N22	L46	E21	Q51,K109,K50	L19	T66
		D35	R118	E31	K115	E21	K68,K109	N32	Q117	N22	L67	E21	K109	E196	K109,K122	Q45	K68	E4	R27	E15	T66,K28,Q72
				D35	R118	E196	K122	D28	Q117,R114	N12	V26	E196	K109,K122	H194	K122	Y29	D48	Y29	K50,D48	A285	K28
				D28	Q117,R114	D195	K123	Y29	L46	D28	R114,S116,K115	D195	K122	D195	K122	E21	K50,K109	D13	K60	R292	Q72,N69
				S25	R114	H194	K123	E21	Q51	E31	K115,R118	H194	K122,Q121	Y29	L46	S24	K109	S25	D48	N22	K68
				S24	R114	H193	K123	S24	K109	D35	R118	N32	R114	S25	D48	N32	R114	N32	R114	D46	N69,K68
				E21	Q51,K50	N206	K123	N199	K115	E196	K122	E31	K115,R118	N32	R114	D28	R114,K115,S116	D28	R114,K115	Q45	K68,N69,Q44
						S24	R114	E31	K115	L198	R120	D28	R114,S116	S24	R114	E31	K115	D35	R118	D195	H105
						D28	R114,S116,K115	D35	R118	D195	K122	D35	R118	D28	R114	D35	R118	E31	R120,K115	H193	K103
						E31	K115,R118	E196	K109,K122	A192	K123			N199	K115,R118	A192	T124	S36	R120	Y29	K50
						D35	R118	D195	K122					E31	R118	N199	S119,R120	D195	K122	D35	R118
								E4	R27					D35	R118	A200	R120	E196	K122	E31	R118
								H194	K122					D13	K60	H194	K122	H194	K122	N199	R122
														N206	T125	E196	K122	A192	K123	Y27	S119
																D195	K122			D28	R118,R114
																				S24	R114
																				N32	R114

Table S7. Polar interactions between TMs in CXCR3_CXCL9 CG-MD.100 ns: R252-V321, 400 ns: Y150-A253, 700 ns: Y150-A253, 800 ns: Y150-A253.

0 ns	TM1	TM2	TM3	TM4	TM5	TM6	TM7
TM1	-	N71-A96, D99	-	-	-	-	Y60-S301
TM2	-	-	-	-	-	-	-
TM3	-	-	-	N132-D186		F135-W268	-
TM4	-	-	-	-	D186-Q219	-	-
TM5	-	-	-	-	-	-	-
TM6	-	-	-	-	-	-	Y271-K300

100 ns	TM1	TM2	TM3	TM4	TM5	TM6	TM7
TM1	-	N71-D99	-	-	-	-	-
TM2	-	-	D99-N134	-	-	-	T105-Y308
TM3	-	-	-	-	-	N134-Y271	R149-Y318
TM4	-	-	-	-	D186-R216, Q219	-	-
TM5	-	-	-	-	-	A222-H272<br>G223-W268	-
TM6	-	-	-	-	-	-	-

200 ns	TM1	TM2	TM3	TM4	TM5	TM6	TM7
TM1	-	Y60-A110	-	-	-	-	-
TM2	-	-	D99-N134<br>L102-N134	H94-W176	-	-	T105-Y308<br>W109-S301
TM3	-	-	-	-	N132-R216, Q219<br>G128-R216	-	S146-Y318
TM4	-	-	-	-	D186-R216, Q219	-	-
TM5	-	-	-	-	-	L225-W268	-
TM6	-	-	-	-	-	-	-

300 ns	TM1	TM2	TM3	TM4	TM5	TM6	TM7
TM1	-	-	-	-	-	-	-
TM2	-	-	D99-N134<br>L102-N134	-	-	-	-
TM3	-	-	-	-	G128-R216<br>N132-L220	F131-Y271<br>Y150-L256	-
TM4	-	-	-	-	D186-R216, Q219	-	-
TM5	-	-	-	-	-	R216-Y271	-
TM6	-	-	-	-	-	-	-

400 ns	TM1	TM2	TM3	TM4	TM5	TM6	TM7
TM1	-	Y60-A110	-	-	-	-	-
TM2	-	-	L102-N134	-	-	-	T105-Y308
TM3	-	-	-	N132-F182, D186	N132-R216	F131-Y271	-
TM4	-	-	-	-	F182-Q219<br>D186-R216	-	-
TM5	-	-	-	-	-	F224-W268	-
TM6	-	-	-	-	-	-	-

500 ns	TM1	TM2	TM3	TM4	TM5	TM6	TM7
TM1	-	N71-D99	-	-	-	-	-
TM2	-	-	D99-N134	-	-	-	T105-Y308
TM3	-	-	-	N132-D186	Y136-F224	-	-
TM4	-	-	-	-	D186-R216, Q219	-	-
TM5	-	-	-	-	-	L225-W268	-
TM6	-	-	-	-	-	-	V274-K300

600 ns	TM1	TM2	TM3	TM4	TM5	TM6	TM7
TM1	-	N71-D99, T100	-	-	-	-	Y60-S301<br>N71-Y308
TM2	-	-	D99-N134<br>L102-N134	-	-	-	L104-Y308
TM3	-	-	-	-	N132-R216, V221<br>Y136-F224	Y150-L256	-
TM4	-	-	-	-	F182-R216<br>D186-R216	-	-
TM5	-	-	-	-	-	G223-H272<br>L225-W268	-
TM6	-	-	-	-	-	-	-

700 ns	TM1	TM2	TM3	TM4	TM5	TM6	TM7
TM1	-	N71-A96	-	-	-	-	-
TM2	-	-	H94-W176	-	-	-	L102-Y308<br>T105-S304
TM3	-	-	-	-	N132-R216<br>Y136-F224	F131-Y271	S146-N314
TM4	-	-	-	-	D186-R216	-	-
TM5	-	-	-	-	-	L225-W268	-
TM6	-	-	-	-	-	-	V274-K300

800 ns	TM1	TM2	TM3	TM4	TM5	TM6	TM7
TM1	-	N71-A96	-	-	-	-	-
TM2	-	-	D99-N134<br>L102-N134	-	-	-	D99-Y308<br>T105-S304
TM3	-	-	-	N132-F182<br>N132-D186	N132-R216, Q219	-	-
TM4	-	-	-	-	D186-R216, Q219	-	-
TM5	-	-	-	-	-	-	-
TM6	-	-	-	-	-	-	-

900 ns	TM1	TM2	TM3	TM4	TM5	TM6	TM7
TM1	-	A75-T100<br>S80-H94	-	-	-	-	-
TM2	-	-	L102-N134	H94-W176	-	-	-
TM3	-	-	-	-	Y136-F224	F131-Y271	N134-Y308
TM4	-	-	-	-	D186-R216,Q219	-	-
TM5	-	-	-	-	-	-	-
TM6	-	-	-	-	-	-	-

1000 ns	TM1	TM2	TM3	TM4	TM5	TM6	TM7
TM1	-	N71-D99	-	-	-	-	-
TM2	-	-	D99-N134	-	-	-	T105-Y308
TM3	-	-	-	-	G128-R216<br>N132-R216, Q219<br>Y136-F224	-	-
TM4	-	-	-	-	D186-R216, Q219	-	-
TM5	-	-	-	-	-	L225-W268<br>P227-W268	-
TM6	-	-	-	-	-	-	-

Table S8. Polar interactions between CXCR3_CXCL10 CG-MD.

0 ns		100 ns		200 ns		300 ns		400 ns		500 ns		600 ns		700 ns		800 ns		900 ns		1000 ns	
R	Q	R	Q	R	Q	R	Q	R	Q	R	Q	R	Q	R	Q	R	Q	R	Q	R	Q
E15	R29	E4	S34,R73	C38	K87	Y27	A81	E31	K87	Y27	K87	D13	R59	S24	K91	E15	R29	P42	K67	E21	K91,V28
E4	R73	E15	R29	S40	K87	S24	L86	D28	K87	E31	K87	E15	R29	E21	K91,Q55	H8	C32	P44	K72	E15	R29
D13	R59	D13	R59	D28	K87	E21	V28	T39	K87	D46	K72,P52	R197	E71,A64	E15	R29	D13	C32,R59	D46	K72	T39	A64
Q9	R37, R59,C32			E21	K91	D7	K80	N50	V28	F47	P52	D195	K47	N22	R29	D7	K80	E4	R43	H8	C30
				D7	K80	D13	R59	D7	K80	Q45	A53	E4	R43	D28	K67	C43	M66	L3	Q38	S6	S79,R59
				D13	R59,I75	L3	R43	S6	K80,R59	N22	V28	S6	N76	T39	K67	E21	K91,V28	E21	K91	D13	R59,K80
				H8	S34	E4	R43	E15	R29	E21	V29,K91	N12	K80	D13	R59	E31	K87	E15	R29	D7	K80
				V10	C30	E15	R29	A14	R29	E15	R29	D7	K80	E4	R43,K80	V115	K72	V10	V28	N12	K80
				E15	R29			D13	R59	D13	R59	E21	K91	E7	K80	D46	K72	D13	C30,R59	M1	S34
								E4	R43	H8	K67	S24	L86			S48	Q55	N12	K80	D28	K67
								E21	K91	E4	E4	T39	K87			S40	T65,K67	D7	K80	S40	K67
								Q45	K91			D28	K87			E4	R43	Y27	L86	C43	M66
								D46	Q55,S54,K72									E31	K87	P44	K72
																		S40	T65	Y27	K87
																				E31	K87
																				E4	R43
																				D46	S54
																				F47	P52

Table S9. Polar interactions between TMs in CXCR3_CXCL10 CG-MD. 0 ns: Y223-H237, Y235-M254, L256-A253 and V258-M254, 200-300 ns: Y150-H337.

0 ns	TM1	TM2	TM3	TM4	TM5	TM6	TM7
TM1	-	N71-T100	-	-	-	-	Y60-S304
TM2	-	-	D99-N134	-	-	-	W109-Y308
TM3	-	-	-	-	-	-	-
TM4	-	-	-	-	D186-Q219	-	-
TM5	-	-	-	-	-	-	-
TM6	-	-	-	-	-	-	F264-H310

100 ns	TM1	TM2	TM3	TM4	TM5	TM6	TM7
TM1	-	-	-	-	-	-	-
TM2	-	-	D99-N134	-	-	-	-
TM3	-	-	-	N132-D186	N132-Q219	-	-
TM4	-	-	-	-	D186-Q219	-	-
TM5	-	-	-	-	-	-	-
TM6	-	-	-	-	-	-	-

200 ns	TM1	TM2	TM3	TM4	TM5	TM6	TM7
TM1	-	-	-	-	-	-	-
TM2	-	-	-	-	-	-	D99-Y308
TM3	-	-	-	N132-F182, D186	-	-	N134-G307<br>L141-Y318
TM4	-	-	-	-	D186-Q219	-	-
TM5	-	-	-	-	-	-	-
TM6	-	-	-	-	-	-	-

300 ns	TM1	TM2	TM3	TM4	TM5	TM6	TM7
TM1	-	-	-	-	-	-	Y60-Y308
TM2	-	-	D99-N134<br>W109-V126	-	-	-	-
TM3	-	-	-	N132-D186	N132-Q219	-	-
TM4	-	-	-	-	D186-Q219	-	-
TM5	-	-	-	-	-	-	-
TM6	-	-	-	-	-	-	-

400 ns	TM1	TM2	TM3	TM4	TM5	TM6	TM7
TM1	-	N71-T100	-	-	-	-	Y60-S304
TM2	-	-	D99-N134	H94-W176	-	-	L102-Y308
TM3	-	-	-	N132-F182, D186	N132-Q219	-	-
TM4	-	-	-	-	D186-Q219	-	-
TM5	-	-	-	-	-	-	Y235-N314
TM6	-	-	-	-	-	-	-

500 ns	TM1	TM2	TM3	TM4	TM5	TM6	TM7
TM1	-	Y60-W109<br>N71-D99	-	-	-	-	-
TM2	-	-	D99-N134	L101-W176	-	-	T105-Y308
TM3	-	-	-	N132-D186	N132-Q219	-	N134-G307
TM4	-	-	-	-	D186-Q219	-	-
TM5	-	-	-	-	-	-	-
TM6	-	-	-	-	-	-	-

600 ns	TM1	TM2	TM3	TM4	TM5	TM6	TM7
TM1	-	N71-L95<br>Y60-W109	-	-	-	-	-
TM2	-	-	D99-N134	H94-W172, W176	-	-	T105-Y308
TM3	-	-	-	N132-D86<br>D148-T171	N132-Q219	-	-
TM4	-	-	-	-	D186-Q219	-	-
TM5	-	-	-	-	-	-	-
TM6	-	-	-	-	-	-	-

700 ns	TM1	TM2	TM3	TM4	TM5	TM6	TM7
TM1	-	N71-D99<br>Y60-W109	-	-	-	-	N71-C311
TM2	-	-	-	-	-	-	D99-Y308<br>L102-Y308
TM3	-	-	-	N132-D186	-	-	-
TM4	-	-	-	-	D186-Q219	-	-
TM5	-	-	-	-	-	F224-W268	-
TM6	-	-	-	-	-	-	-

800 ns	TM1	TM2	TM3	TM4	TM5	TM6	TM7
TM1	-	Y60-T105	-	-	-	-	-
TM2	-	-	D99-N134	-	-	-	D99-Y308
TM3	-	-	-	N132-D186	N132-Q219	-	N134G307<br>R150Y318
TM4	-	-	-	-	D186-Q219	-	-
TM5	-	-	-	-	-	-	-
TM6	-	-	-	-	-	-	F264-H310

900 ns	TM1	TM2	TM3	TM4	TM5	TM6	TM7
TM1	-	N71-D99<br>Y60-T105	-	-	-	-	-
TM2	-	-	D99-N134	H94-W176	-	-	L102-Y308
TM3	-	-	-	-	-	-	-
TM4	-	-	-	-	-	-	-
TM5	-	-	-	-	-	-	-
TM6	-	-	-	-	-	-	-

1000 ns	TM1	TM2	TM3	TM4	TM5	TM6	TM7
TM1	-	Y60-T105	-	-	-	-	-
TM2	-	-	D99-N134	H94-W176	-	-	L102-308
TM3	-	-	-	-	Y136-Q219	-	-
TM4	-	-	-	-	D186-Q219	-	-
TM5	-	-	-	-	-	-	-
TM6	-	-	-	-	-	-	-

Table S10. Polar interactions between CXCR3_CXCL11 CG-MD.

0 ns		100 ns		200 ns		300 ns		400 ns		500 ns		600 ns		700 ns		800 ns		900 ns		1000 ns	
R	Q	R	Q	R	Q	R	Q	R	QK	R	Q	R	Q	R	Q	R	Q	R	Q	R	Q
N22	A48	D7	K26	D46	K80	E21	R83	E15	K38	S6	F25,K26	D7	K26	E4	R27	E15	N55,K47	V2	R27	D13	R29
A18	S49	E4	R27,G28	E293	K88	S25	R83	D13	R29	M1	K26	E4	K26,R27	D13	R29	E4	R27	E4	R27	R197	E90,V89
D46	K87	S6	R29	E15	K70	V5	R29	E4	R27	D7	K26	L3	R27	E15	K70	D7	G28,R27,K26	D7	V37,K38	S6	K38
L11	Q72	D13	R29	H8	G28,K2 6	D13	R29	D7	K26	E4	R27	E21	R83	H194	R91	A18	R83	E15	K47,K41	D7	K38
V2	M24	N289	K88,K47	Q9	K26	E4	R27	N50	K78	E15	K70	A18	N56	D195	R91	E21	R83	D13	R29	S40	R91
E33	K92	E15	K70	D7	K26,R2 7	S6	R27, G28	F47	K80	D13	R27,R29	E15	Y52,S49, K70	E196	R91	E31	K92	E21	R83	E33	R91
Q9	R29	D46	K80	E4	R27	D7	K26	E21	R83	A18	S54	H194	R91	E33	R91	Y27	K92	D52	S79	P41	R91
D13	R29,N56	E21	R83	V5	R29	D195	R91	N289	E90	E21	N56,R83	E33	R91	R197	E90	R197	E90	N50	S79,K78	S26	N93
		S25	R83	D13	R29	E33	R91	R197	E90	S25	R83	R197	E90	D7	K26,K47	D13	R29	F47	Q81	A18	K59
		E293	K87	S34	R91	R197	E90	L198	V89	Q45	R83	N199	K88	E31	K92	E293	K88	D46	K87	E21	R83
		Y29	R91	H194	R91	N289	E90, K88	T201	K87	D46	R83,K87	L198	K87	N22	S54	T201	K87	R197	E90	E15	K47
		C290	K87	E196	R91	E293	K88	N199	K88	A200	K87	Q45	K87	E21	R83	N199	K87	E31	K92	F47	Q81
		S26	R91,K92	E33	R91	D46	K87, K80	E293	K88	V118	K87	D46	A82	D46	A82,K87	E33	R91	G30	K92	Q45	K87
		R197	E90	G30	R91	Q45	K80	E33	R91	N199	K88	F47	Q81	E293	K88	D46	K87	E196	R91	D297	Q81
		Y27	K92	E31	K92	E15	K70	G30	R91	N289	K88	S48	S79	D52	K78	S48	K78	E33	R91	E4	K26
		E31	K92	S26	N93,E9 0	S26	K92			N50	K78	D52	S79,K78							E293	K88
				R197	E90	E31	K92			R197	E90,V89	Y27	K92							N289	K88
				Y29	K87,i86					N22	I85	D13	R29							A287	T65
				N199	K87					E31	K92									N50	K78
				S25	R83					E33	R91										
				E21	I50,r83					S34	R91										
				C43	Q81					H194	R91										
										S48	Q81										

Table S11. Polar interactions between TMs in CXCR3_CXCL11 CG-MD. 500 ns: Y150-C234, H237, 600: R252-F320, 700 ns: R252-F320, 800 ns: R252-F320.

0 ns	TM1	TM2	TM3	TM4	TM5	TM6	TM7
TM1	-	-	-	-	-	-	-
TM2	-	-	D99-N134	-	-	-	-
TM3	-	-	-	N132-D186	-	-	-
TM4	-	-	-	-	D186-Q219	-	-
TM5	-	-	-	-	-	-	-
TM6	-	-	-	-	-	-	-

100 ns	TM1	TM2	TM3	TM4	TM5	TM6	TM7
TM1	-	-	-	-	-	-	-
TM2	-	-	D99-N134	-	-	-	-
TM3	-	-	-	N132-D186	-	-	-
TM4	-	-	-	-	D186-Q219	-	-
TM5	-	-	-	-	-	-	-
TM6	-	-	-	-	-	-	-

200 ns	TM1	TM2	TM3	TM4	TM5	TM6	TM7
TM1	-	-	-	-	-	-	Y60-S301
TM2	-	-	D99-N134	H94-W176	-	-	D99-Y308
TM3	-	-	-	-	A139-A222<br>Y150-V230	N134-Y271<br>G138-W268	-
TM4	-	-	-	-	D186-Q219	-	-
TM5	-	-	-	-	-	G223-W268	-
TM6	-	-	-	-	-	-	-

300 ns	TM1	TM2	TM3	TM4	TM5	TM6	TM7
TM1	-	-	-	-	-	-	Y60-S301
TM2	-	-	-	-	-	-	-
TM3	-	-	-	N132-D186, F182	-	G138-W268	-
TM4	-	-	-	-	D186-Q219	-	-
TM5	-	-	-	-	-	-	-
TM6	-	-	-	-	-	-	-

400 ns	TM1	TM2	TM3	TM4	TM5	TM6	TM7
TM1	-	-	-	-	-	-	Y60-S301
TM2	-	-	D99-N134	-	-	-	-
TM3	-	-	-	N132-D186	-	-	-
TM4	-	-	-	-	D186-Q219	-	-
TM5	-	-	-	-	-	-	-
TM6	-	-	-	-	-	-	-

500 ns	TM1	TM2	TM3	TM4	TM5	TM6	TM7
TM1	-	N71-T100	-	-	-	-	-
TM2	-	-	D99-N134	H94-W176	-	-	D99-Y308<br>L102-Y308
TM3	-	-	-	N132-D186	Y150-C234	N134-Y271<br>G138-W268	-
TM4	-	-	-	-	-	-	-
TM5	-	-	-	-	-	-	-
TM6	-	-	-	-	-	-	-

600 ns	TM1	TM2	TM3	TM4	TM5	TM6	TM7
TM1	-	-	-	-	-	-	Y60-S301
TM2	-	-	D99-N134	-	-	-	L92-318<br>D99-Y308<br>L102-Y308<br>W109-S304
TM3	-	-	-	N138-F182	Y136-L218<br>Y150-Y233, C234	G138-W268	-
TM4	-	-	-	-	D186-Q219	-	-
TM5	-	-	-	-	-	-	-
TM6	-	-	-	-	-	-	-

700 ns	TM1	TM2	TM3	TM4	TM5	TM6	TM7
TM1	-	N71-D99<br>S80-H94	-	-	-	-	-
TM2	-	-	D99-N134	H94-W176	-	-	D99-Y308<br>L102Y308<br>L104-Y308<br>W109-S304
TM3	-	-	-	-	Y150-Y233	G138-W268	-
TM4	-	-	-	-	R186-Q219	-	-
TM5	-	-	-	-	-	-	-
TM6	-	-	-	-	-	-	Y271-L306

800 ns	TM1	TM2	TM3	TM4	TM5	TM6	TM7
TM1	-	-	-	-	-	-	Y600-S301
TM2	-	-	D99-N134	H94-W176	-	-	D99-Y308
TM3	-	-	-	N132-D186	-	G138-W268	-
TM4	-	-	-	-	-	-	-
TM5	-	-	-	-	-	-	-
TM6	-	-	-	-	-	-	-

900 ns	TM1	TM2	TM3	TM4	TM5	TM6	TM7
TM1	-	-	-	-	-	-	-
TM2	-	-	D99-N134	H94-W176	-	-	D99-Y308
TM3	-	-	-	N132-D186	-	N132-Y271<br>G138-W268	-
TM4	-	-	-	-	D186-Q219	-	-
TM5	-	-	-	-	-	-	-
TM6	-	-	-	-	-	-	-

1000 ns	TM1	TM2	TM3	TM4	TM5	TM6	TM7
TM1	-	N71-D99	-	-	-	-	-
TM2	-	-	D99-N134	-	-	-	D99-Y308
TM3	-	-	-	N132-D186	Y136-L218	-	-
TM4	-	-	-	-	D186-Q219	-	-
TM5	-	-	-	-	-	-	-
TM6	-	-	-	-	-	-	-

Table S12. Polar interactions between TMs in CXCR3/GP complex 1 μs. The arginine cage is present at 1000 ns and R149 interacts with TM5 and TM7. The residue R149 is not oriented to GP in any frame of simulation.

0 ns	TM1	TM2	TM3	TM4	TM5	TM6	TM7
TM1	-	-	-	-	-	-	Y60-S301
TM2	-	-	D99-N134	-	-	-	-
TM3	-	-	-	-	Y136-L218<br>N132-Q219	-	A127,F131-Y308
TM4	-	-	-	-	D186-Q219	-	D186-K300
TM5	-	-	-	-	-	-	-
TM6	-	-	-	-	-	-	L256-Y318

100 ns	TM1	TM2	TM3	TM4	TM5	TM6	TM7
TM1	-	N71-T100	-	-	-	-	-
TM2	-	-	-	-	-	-	-
TM3	-	-	-	-	-	N132-Y271	A127-S304
TM4	-	-	-	-	D186-Q219	-	D186-K300
TM5	-	-	-	-	-	-	-
TM6	-	-	-	-	-	-	-

200 ns	TM1	TM2	TM3	TM4	TM5	TM6	TM7
TM1	-	-	-	-	-	-	-
TM2	-	-	D99-N134	-	-	-	-
TM3	-	-	-	N132-D186	Y136-L218	-	I145,R149-Y318
TM4	-	-	-	-	D186-Q219	-	D186-K300
TM5	-	-	-	-	-	-	-
TM6	-	-	-	-	-	-	-

300 ns	TM1	TM2	TM3	TM4	TM5	TM6	TM7
TM1	-	-	-	-	-	-	-
TM2	-	-	D99-N134	-	-	-	-
TM3	-	-	-	-	Y136-L218<br>S146-P227, Y235	N132-Y271	A127-S304<br>S146-Y318<br>R149-Y318
TM4	-	-	-	-	D186-Q219	-	D186-K300
TM5	-	-	-	-	-	R216-V275	-
TM6	-	-	-	-	-	-	Y271-K300<br>A263-N314

400 ns	TM1	TM2	TM3	TM4	TM5	TM6	TM7
TM1	-	-	-	-	-	-	-
TM2	-	-	D99-N134	-	-	-	-
TM3	-	-	-	-	Y136-L218	G128, N132-Y271	L130-Y308<br>I145-Y318
TM4	-	-	-	-	-	-	D186-K300
TM5	-	-	-	-	-	L220-H272	Q219-K300<br>Y235-Y318
TM6	-	-	-	-	-	-	Y271-K300<br>V260-N314

500 ns	TM1	TM2	TM3	TM4	TM5	TM6	TM7
TM1	-	-	-	-	-	-	-
TM2	-	-	D99-N134	-	-	-	-
TM3	-	-	-	N132-F186, D186	S146, Y150-Y235<br>Y136-L218	G128, N132-Y271	L130-S304<br>N134-Y308<br>I145, R149-Y318
TM4	-	-	-	-	D186-Q219	-	D186-K300
TM5	-	-	-	-	-	-	-
TM6	-	-	-	-	-	-	-

600 ns	TM1	TM2	TM3	TM4	TM5	TM6	TM7
TM1	-	-	-	-	-	-	N71-C312
TM2	-	-	-	-	-	-	-
TM3	-	-	-	-	Y136-L218	F131-Y271	A127-S304
TM4	-	-	-	-	-	-	D186-K300
TM5	-	-	-	-	-	-	Q219-K300
TM6	-	-	-	-	-	-	-

700 ns	TM1	TM2	TM3	TM4	TM5	TM6	TM7
TM1	-	-	-	-	-	-	-
TM2	-	-	D99-N134	-	-	-	-
TM3	-	-	-	-	N132-Q219<br>Y136-L218<br>Y150-C234	A126-Y271	A126-S304<br>R149-Y318
TM4	-	-	-	-	D186-Q219	-	D186-K300
TM5	-	-	-	-	-	-	-
TM6	-	-	-	-	-	-	A263-N314

800 ns	TM1	TM2	TM3	TM4	TM5	TM6	TM7
TM1	-	-	-	-	-	-	-
TM2	-	-	D99-N134	-	-	-	-
TM3	-	-	-	N132-F182	Y136-L218	-	A126-S304<br>N134-Y308
TM4	-	-	-	-	-	-	D186-K300
TM5	-	-	-	-	-	R216-V275<br>Q219-Y271	-
TM6	-	-	-	-	-	-	-

900 ns	TM1	TM2	TM3	TM4	TM5	TM6	TM7
TM1	-	-	-	-	-	-	N71-C312
TM2	-	-	D99-N134	-	-	-	D99-Y308
TM3	-	-	-	-	N132-F181	N132-Q219<br>R149-Y235	N134-Y308<br>R149-V231
TM4	-	-	-	-	-	-	D186-K300
TM5	-	-	-	-	-	Q219-Y271	-
TM6	-	-	-	-	-	-	A263-H310

1000 ns	TM1	TM2	TM3	TM4	TM5	TM6	TM7
TM1	-	-	-	-	-	-	-
TM2	-	-	D99-N134	-	-	-	-
TM3	-	-	-	-	-	-	A126-S304<br>L130-Y308<br>I145-Y318<br>R149-Y318
TM4	-	-	-	-	D186-Q219	-	D186-K300
TM5	-	-	-	-	-	Q219-Y271<br>L220-H272	-
TM6	-	-	-	-	-	-	-

Table S13. Polar interactions between CXCR3_CXCL10 250 ns AA-MD.

0 ns		25 ns		50 ns		75 ns		100 ns		125 ns		150 ns		175 ns		200 ns		225 ns		250 ns	
R	Q	R	Q	R	Q	R	Q	R	Q	R	Q	R	Q	R	Q	R	Q	R	Q	R	Q
E31	K87	P41	K67	D13	K80	S40	T65, K67	S24	K91	D13	K83	E4	N37	S24	K91	A18	Q55	E21	R59	E21	R59
E4	R43	S40	K67	S48	Q55	P41	K67	D28	K91	E21	K91	A14	C30	D28	K91	E21	R59	A14	C30	D13	K87
D13	R59	C43	K67	S40	K67	C43	K67	S40	A64, K67	*Y27	K87	E21	R59, K91	P41	K67	D7	K83	H8	N37	D46	K67
H8	R59, E78, N76	D13	K80	P41	K67	E21	K91	E21	R59			C43	K67	L3	N37	D13	K83			A14	C30
S40	A64	E21	K91	C43	K67			*Y27	K87			S40	K67	D7	R29	P41	K67			S6	S36, Q38
D28	K67	NAG32	K87	*Y27	K87							*Y27	K88	E15	R29	*Y27	K87				
S24	K67													E21	R59						
Q116	K72																				
E21	K91																				
N22	V28																				
S48	V28, R29																				
E15	R29																				

Table S14. Polar interactions between TMs in CXCR3_CXCL10 250 ns AA-MD.

0 ns	TM1	TM2	TM3	TM4	TM5	TM6	TM7
TM1	-	N71-D99<br>S80-H94	-	-	-	-	W71-C311
TM2	-	-	-	V97-W176	-	-	T105-S304
TM3	-	-	-	N132-D186	N132-219	-	-
TM4	-	-	-	-	-	-	-
TM5	-	-	-	-	-	-	-
TM6	-	-	-	-	-	-	-

25 ns	TM1	TM2	TM3	TM4	TM5	TM6	TM7
TM1	-	N71-D99	-	-	-	-	-
TM2	-	-	-	-	-	-	-
TM3	-	-	-	N132-F182, D186	-	-	N134-C311
TM4	-	-	-	-	-	-	-
TM5	-	-	-	-	-	-	-
TM6	-	-	-	-	-	-	-

50 ns	TM1	TM2	TM3	TM4	TM5	TM6	TM7
TM1	-	N71-D99	-	-	-	-	-
TM2	-	-	-	-	-	-	-
TM3	-	-	-	N132-D186	S146-Y235	-	A137-N314
TM4	-	-	-	-	-	-	-
TM5	-	-	-	-	-	R216-Y271	-
TM6	-	-	-	-	-	-	-

75 ns	TM1	TM2	TM3	TM4	TM5	TM6	TM7
TM1	-	-	-	-	-	-	-
TM2	-	-	-	-	-	-	-
TM3	-	-	-	N132-D186	S146-<br>Y235	N134-W268	A137-N314<br>R149-Y318
TM4	-	-	-	-	-	-	-
TM5	-	-	-	-	-	Q219-Y271	-
TM6	-	-	-	-	-	-	-

100 ns	TM1	TM2	TM3	TM4	TM5	TM6	TM7
TM1	-	N71-D99	-	-	-	-	-
TM2	-	-	-	-	-	-	-
TM3	-	-	-	N132-F182	-	-	A137-N314
TM4	-	-	-	-	-	-	-
TM5	-	-	-	-	-	R216-Y271	-
TM6	-	-	-	-	-	-	-

125 ns	TM1	TM2	TM3	TM4	TM5	TM6	TM7
TM1	-	N71-D99	-	-	-	-	-
TM2	-	-	-	-	-	-	-
TM3	-	-	-	N132-D186	-	N134-W268	A137-N314
TM4	-	-	-	-	-	-	-
TM5	-	-	-	-	-	R216-Y271	-
TM6	-	-	-	-	-	-	-

150 ns	TM1	TM2	TM3	TM4	TM5	TM6	TM7
TM1	-	-	-	-	-	-	-
TM2	-	-	-	-	-	-	-
TM3	-	-	-	N132-D186	-	-	-
TM4	-	-	-	-	-	-	-
TM5	-	-	-	-	-	-	-
TM6	-	-	-	-	-	-	-

175 ns	TM1	TM2	TM3	TM4	TM5	TM6	TM7
TM1	-	-	-	-	-	-	-
TM2	-	-	-	-	-	-	-
TM3	-	-	-	N132-D186	-	N134-W268	A137-N314
TM4	-	-	-	-	-	-	-
TM5	-	-	-	-	-	-	-
TM6	-	-	-	-	-	-	-

200 ns	TM1	TM2	TM3	TM4	TM5	TM6	TM7
TM1	-	N71-<br>D99-	-	-	-	-	-
TM2	-	-	-	-	-	-	W109-S302
TM3	-	-	-	N132-D186	-	N134-W268	A137-N314
TM4	-	-	-	-	-	-	-
TM5	-	-	-	-	-	-	-
TM6	-	-	-	-	-	-	-

225 ns	TM1	TM2	TM3	TM4	TM5	TM6	TM7
TM1	-	A76-D99	-	-	-	-	-
TM2	-	-	-	-	-	-	-
TM3	-	-	-	N132-F182	-	N134-W268	A137-N314
TM4	-	-	-	-	-	-	-
TM5	-	-	-	-	-	-	-
TM6	-	-	-	-	-	-	-

250 ns	TM1	TM2	TM3	TM4	TM5	TM6	TM7
TM1	-	-	-	-	-	-	-
TM2	-	-	-	-	-	-	W109-S302
TM3	-	-	-	N132- F182, D186	N132-Q219	N134-W268	A137-N314
TM4	-	-	-	-	-	-	-
TM5	-	-	-	-	-	Q219-Y271	-
TM6	-	-	-	-	-	-	-

Table S15. Polar interactions between CXCR3_alpha subunit 250 ns AA-MD.

0 ns		25 ns		50 ns		75 ns		100 ns		125 ns		150 ns		175 ns		200 ns		225 ns		250 ns	
R	A	R	A	R	A	R	A	R	A	R	A	R	A	R	A	R	A	R	A	R	A
R250	D337	R250	D337	R342	D25	A84	H195	R342	E27	S364	K209	R353	D19	R149	D193	T83	E32	R149	D193	A84	R31
R149	D193	E327	R31	R82	T190	R353	D19	Y365	L353,W258	Y365	L353	T157	K192	R353	D19	R82	E24	R342	E27	T83	R31
L79	E24, R23	W330	E24	T88	D193	R342	D25	Q341	D25	R353	D19	R149	D193	L159	Q333	S80	R25	R161	H56	D89	R31
R342	R23, D25	R342	D25	Y365	G352, W258	R352	C2	R335	D25	T83	H195	R342	E27	Q158	Q333	W330	R23	W330	R23	R249	D341
W330	E37	S87	D193	R162	Q333	W330	E24	R353	D19	R342	D25, E27	S366	F354	R161	Q51, T329	R353	D19	R82	E24	R82	E24
R353	D19	T88	D193	E327	R31	S87	D193	R162	Q333	Y160	Q333	W330	R23	W330	R23	R342	E27	L159	Q333	W330	R23
Y265	Q217	Y365	W258, L353	C338	K28	Y365	G217, W258	Y160	Q333	R161	Q51, Q333	R161	I54	R82	E24	T157	D337	T83	R31	R149	D193
R249	D341	R161	H56	R353	D19			A84	T190	R149	D193	Y365	G217, W258, L353	R342	E27	R161	Q51, K53, T329	D89	R31	Y365	L353
		R353	D19	R335	R23			S87	D193	S366	W258	E327	R31	S366	F354	R149	D193	A84	W258	R342	E27
								E327	R31					Y365	G217	Y365	G217, W258	Y365		Q158	K330
																				L159	Q333
